# Bispecific Antibody Inhalation Therapy for Redirecting Stem Cells from the Lungs to Repair Heart Injury

**DOI:** 10.1002/advs.202002127

**Published:** 2020-11-19

**Authors:** Mengrui Liu, Halle Lutz, Dashuai Zhu, Ke Huang, Zhenhua Li, Phuong‐Uyen C. Dinh, Junqing Gao, Yi Zhang, Ke Cheng

**Affiliations:** ^1^ Department of Molecular Biomedical Sciences North Carolina State University North Carolina USA; ^2^ Joint Department of Biomedical Engineering University of North Carolina at Chapel Hill and North Carolina State University North Carolina USA; ^3^ Comparative Medicine Institute North Carolina State University North Carolina USA; ^4^ Department of Cardiology Putuo Hospital Shanghai University of Traditional Chinese Medicine Shanghai China; ^5^ Department of Cardiology Shanghai Tenth People's Hospital Tongji University School of Medicine Shanghai China

**Keywords:** bispecific antibodies, heart repair, hematopoietic stem cells, inhalation delivery, myocardial infarction

## Abstract

Stem cell therapy is a promising strategy for cardiac repair. However, clinical efficacy is hampered by poor cell engraftment and the elusive repair mechanisms of the transplanted stem cells. The lung is a reservoir of hematopoietic stem cells (HSCs) and a major biogenesis site for platelets. A strategy is sought to redirect lung resident stem cells to the injured heart for therapeutic repair after myocardial infarction (MI). To achieve this goal, CD34‐CD42b platelet‐targeting bispecific antibodies (PT‐BsAbs) are designed to simultaneously recognize HSCs (via CD34) and platelets (via CD42b). After inhalation delivery, PT‐BsAbs reach the lungs and conjoined HSCs and platelets. Due to the innate injury‐finding ability of platelets, PT‐BsAbs guide lung HSCs to the injured heart after MI. The redirected HSCs promote endogenous repair, leading to increased cardiac function. The repair mechanism involves angiomyogenesis and inflammation modulation. In addition, the inhalation route is superior to the intravenous route to deliver PT‐BsAbs in terms of the HSCs’ homing ability and therapeutic benefits. This work demonstrates that this novel inhalable antibody therapy, which harnesses platelets derived from the lungs, contributes to potent stem cell redirection and heart repair. This strategy is safe and effective in a mouse model of MI.

## Graphical abstract

Platelet‐targeting bispecific antibodies (PT‐BsAbs) are designed to recognize hematopoietic stem cells (HSCs) and platelets simultaneously in the lung. Due to the innate injury‐finding ability of platelets, inhaled PT‐BsAbs guide lung HSCs to the injured heart for cardiac repair.

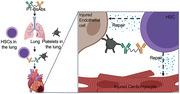

## 1 Introduction

Acute myocardial infarction (MI) is a global leading cause of death.^[^ ^1^ ^]^ The difficulty of MI therapy lies in the low turnover rate of cardiomyocytes (0.3% to 1%),^[^ ^2^ ^]^ and the remodeling of the extracellular matrix, which accelerates cardiac fibrosis and heart failure.^[^ ^3^, ^4^ ^]^ Several cell types, namely cardiac progenitor cells, cardiosphere‐derived cells, endothelial progenitor cells, mesenchymal stem cells (MSCs), and bone marrow mononuclear cells, have been tested in clinical trials but with disappointing results.^[^ ^5^, ^6^, ^7^ ^]^ Endogenous stem cells have become one of the most important objects of research for cardiomyocyte regeneration over the last two decades. In fact, as the body's own repair mechanism, endogenous stem cells from the bone marrow are recruited into the heart for repair after MI.^[^ ^8^ ^]^ However, the stem cells’ lack of targeting hinders their ability to accumulate in the MI area. Even with the bone marrow stimulating agent granulocyte colony‐stimulating factor (G‐CSF), the endogenous repair is not sufficient to offset the injury.^[^ ^9^ ^]^ In our previous studies, we demonstrated that MI biomarkers could be utilized to guide infused or endogenous stem cells to the injured heart.^[^ ^10^, ^11^ ^]^ Furthermore, ischemic heart injuries, such as MI, have been shown to induce vascular damage. This damage can expose components of the subendothelial matrix, such as von Willebrand factor (vWF), collagen, and fibronectin, which leads to platelet recruitment. Platelets can directly bind to injured endothelial cells through receptors expressed on the cells’ surfaces. For example, glycoprotein (GP) VI, GPIV, GPIb, and GPIX contribute to the recruiting of platelets.^[^ ^12^, ^13^ ^]^ This fact makes platelets a robust platform for injury targeting. By taking advantage of the MI injury‐homing ability of platelets,^[^ ^14^, ^15^ ^]^ we can target stem cells to the MI area.^[^ ^14^, ^16^ ^]^ Nevertheless, this strategy has a caveat. Most of the body's endogenous stem cells are produced in the bone marrow but these cells are difficult to access. Moreover, while there are circulating stem cells in the blood stream, there aren't that many of them to attract.

**Figure 1:**
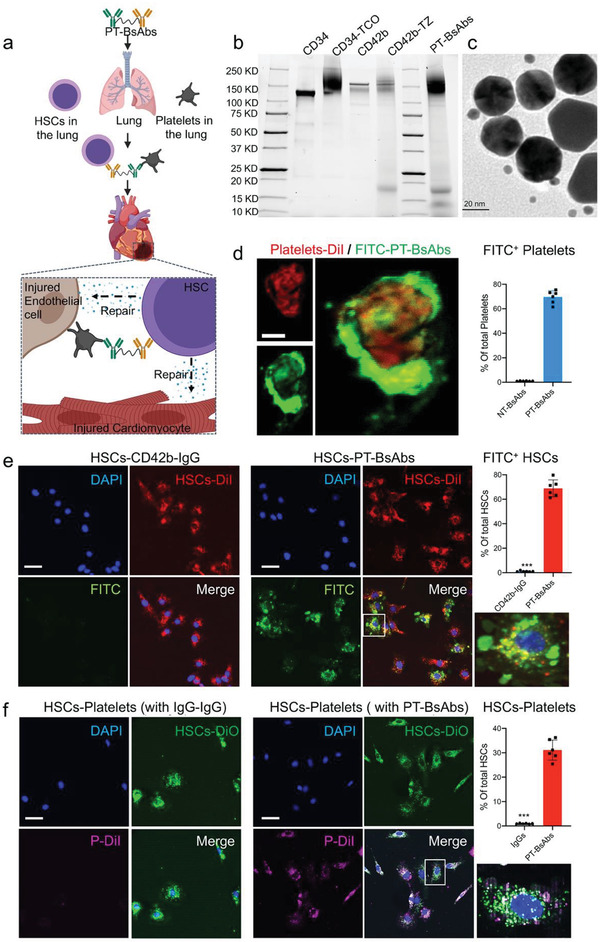
Fabrication and characterization of PT‐BsAbs. a) Scheme illustrating PT‐BsAbs‐mediated HSCs‐targeting and heart repair. b) SDS‐PAGE results of CD34, CD42b, semi‐products (CD34‐TCO, CD42b‐TZ) and PT‐BsAbs. c) Transmission electron microscopy (TEM) image confirming the linkage of CD34 (rat) and CD42b (mouse) antibodies (labeled with anti‐rat gold nanoparticles (25 nm) and anti‐mouse gold nanoparticles (5 nm), respectively) to form the final product PT‐BsAbs. Scale Bar, 20 µm. d) Confocal images showing the binding efficiency of PT‐BsAbs (but not NT‐BsAbs) (green) to a DiI‐labeled platelet (red). Bound PT‐BsAbs were detected with FITC‐labeled anti CD34 antibodies. Scale Bar, 1 µm. e) Confocal images indicating the binding efficiency of PT‐BsAbs (but not CD42b‐IgG) (green) to DiI‐labeled HSCs (red). HSC nuclei were counter‐stained with DAPI. Attached PT‐BsAbs were detected with FITC‐labeled anti‐CD42b antibodies. Scale Bar, 10 µm. f) Confocal images showing the linkage of DiI labeled‐platelets (red) and DiO‐labeled‐HSCs (green) with PT‐BsAbs (but not with IgG‐IgG), HSC nuclear was stained with DAPI. Scale Bar, 10 µm.

Recently, the lung has been recognized as a new hematopoietic organ and as a new site for platelet biogenesis.^[^ ^17^ ^]^ Since the lungs contain both the cells (HSCs) and the navigator cells (platelets) at a high concentration, we sought to develop a strategy to link those two types of cells in situ. That way, the platelets can piggy‐back ride on the hematopoietic stem cells (HSCs) to the infarct site for repair. To do this, we designed a targeting bispecific agent, namely, the platelet‐targeting bispecific antibody (PT‐BsAb). Essentially, PT‐BsAbs are CD34 (HSC binding) and CD42b (platelet binding)‐linked BsAbs. In addition, unlike previous strategies, we can potentially use inhalation delivery as a safe, non‐invasive, and convenient route to administer these therapeutic BsAbs. Our therapeutic concept is displayed in **Figure** **1**a.

In a mouse model of MI injury, we tested the proof‐of‐concept for inhalation delivery of PT‐BsAbs. We tested whether inhaled PT‐BsAbs could link HSCs with platelets and redirect HSCs to the injured heart. The treatment effects and the underlying mechanisms were investigated. In addition, we benchmarked the inhalation delivery method against the intravenous infusion of the same PT‐BsAb therapeutic.

## 2 Results

### 2.1 The Lung is a Reservoir for Hematopoietic Stem Cells

Even though the bone marrow is the primary source of HSCs, these cells were recently reported to migrate to, and not be entrapped by the lungs, which also harbor platelets.^[^ ^18^, ^19^, ^20^ ^]^ We confirmed the presence of HSCs in the lungs with CD34 marker using cell sorting and compared the percentage of HSCs in the bone marrow to that of the lung. Results revealed that HSCs accounted for about 1.7% (Figure S1, Supporting Information) of bone marrow‐derived cells, consistent with a published literature.^[^ ^21^ ^]^ In contrast, CD34^+^ HSCs accounted for 6.1% of total cells in the lungs.

### 2.2 Fabrication and Characterization of Platelet‐Targeting Bispecific Antibodies

PT‐BsAbs were synthesized using a biorthogonal chemical reaction (Figure S2, Supporting Information). With a high specific property, trans‐cyclooctene (TCO) and tetrazine (TZ) polyethylene glycol (PEG) derivatives were chosen as reagents, which could react with each other and form strong covalent bonds quickly in physiological conditions. In brief, CD42b, the platelet binding motif, was first modified with TZ‐PEG‐NHS to form targeting segments (CD42b‐TZ). Meanwhile, TCO‐PEG‐NHS was conjugated to CD34 (CD34^+^ HSCs binding ligands), yielding the pre‐capturing groups (CD34‐TCO). Finally, PT‐BsAbs were constructed via TCO and TZ conjugation. SDS‐PAGE was performed to confirm the successful conjugation (Figure 1b). Compared to pure antibodies, polymer‐conjugated antibodies presented discrete free protein bands in SDS‐PAGE gels. IgG‐IgG, CD42b‐IgG, and non‐targeting BsAbs (NT‐BsAbs, CD34‐IgG) were also constructed and characterized as control groups (Figure S3, Supporting Information). Moreover, distinct gold nanoparticle‐labeled secondary antibodies were used to demonstrate the structure of PT‐BsAbs, since these antibodies were from different hosts. Results revealed that 5 nm gold nanoparticles (anti‐mouse, labeling CD42b) distributed around 25 nm gold nanoparticles (anti‐rat, labeling CD34) (Figure 1c), which confirmed the successful combination between CD34 and CD42b in PT‐BsAbs.

### 2.3 Platelet‐Targeting Bispecific Antibodies Bind to Hematopoietic Stem Cells and Platelets

PT‐BsAbs were expected to capture CD34^+^ HSCs and target the MI site with the homing ability of bound CD42b. Therefore, the binding efficiency of PT‐BsAbs played a critical role in their applications. We isolated platelets from mouse blood. Platelets were first incubated with PT‐BsAbs (CD34‐CD42b), followed by incubation with FITC‐labeled anti‐CD34 antibody. Cell‐bound PT‐BsAbs were detected around platelets. Confocal microscopy revealed the high binding capability of PT‐BsAbs (green) to DiI‐labeled platelets (red) (Figure 1d). In addition, confocal microscopy results revealed the high binding capability of PT‐BsAbs (green) to DiI‐labeled HSCs (red) (Figure 1e).

### 2.4 Platelet‐Targeting Bispecific Antibodies Conjoin Hematopoietic Stem Cells with Platelets

With the presence of PT‐BsAbs, a great number of DiI‐labeled platelets were combined with DiO‐labeled HSCs, forming HSCs‐platelets aggregates (HSC‐PLT) (Figure 1f, 31.1%). In turn, platelets were hardly attached to HSCs with IgG‐IgG. Flow cytometry further quantified the binding efficiency (Figure S4, Supporting Information). After being incubated with DyLight 633‐labeled PT‐BsAbs, 65.3% of HSCs were bound to PT‐BsAbs. 37.4% of these cells were further able to form HSCs‐PLT. These results demonstrated the potential utility of PT‐BsAbs, which were not only able to capture CD34^+^ HSCs but also bind platelets via CD42b.

### 2.5 Inhaled Platelet‐Targeting Bispecific Antibodies First Reside in the Lungs and then Migrate to the Heart

**Figure 2:**
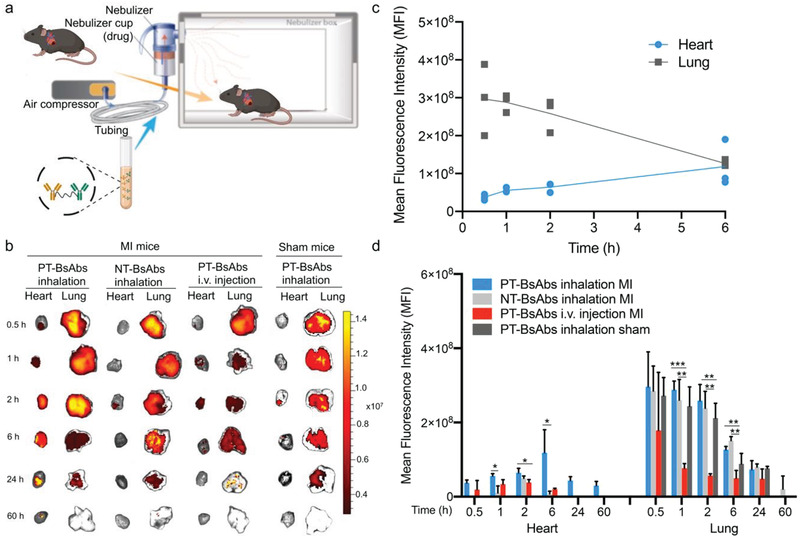
Biodistribution of inhaled PT‐BsAbs assessed by fluorescent imaging. a) Schematic showing the inhalation system. b) Ex vivo imaging to evaluate the distribution of PT‐BsAbs or NT‐BsAb after inhalation, PT‐BsAbs after i.v. injection in MI mice, and PT‐BsAbs after inhalation in sham mice at different time points. c) Time‐course quantification of fluorescence signals from heart and lung tissue of MI mice treated with PT‐BsAbs via inhalation. d) Time‐course quantification of fluorescence signals from the heart and lung of the mice. *N* = 3.

**Figure 3:**
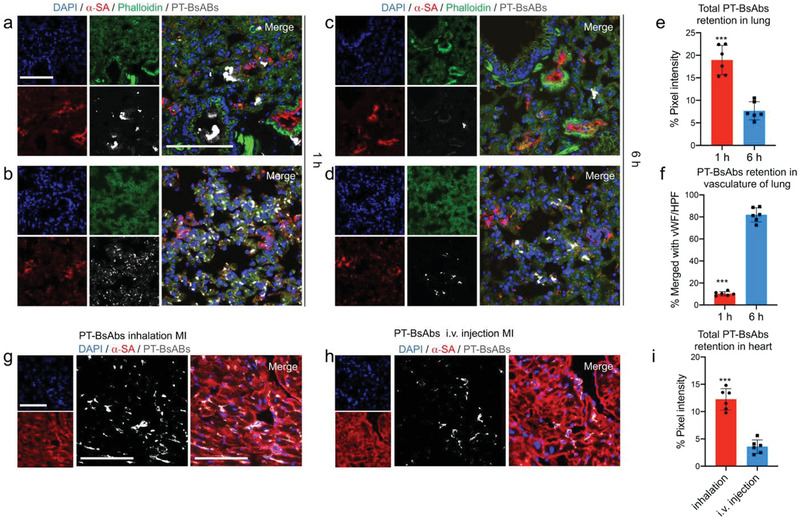
Biodistribution of inhaled PT‐BsAbs assessed by histology. a–d) Representative confocal images showing distribution of PT‐BsAbs in the bronchi and parenchyma of MI mice, respectively, a,b) 1 h and c,d) 6 h after inhalation. PT‐BsAbs were pre‐labeled with DyLight 633 (gray). Lung cells were stained with AF 594‐labeled Phalloidin antibody (green), lung vasculature was stained by von Willebrand Factor (vWF) antibody (red); nuclei were stained with DAPI (blue). Scale bar, 100 µm. e) Quantification of PT‐BsAbs in MI mice in the lung 1 h and 6 h after inhalation, respectively. f) Time‐course quantification results of PT‐BsAbs in MI mice in vasculature of lung 1 h and 6 h after inhalation, respectively. Confocal imaging revealing the PT‐BsAbs retention in the border zone of MI heart 6 h after g) inhalation or h) i.v. injection. Cardiomyocytes were stained with alpha sarcomeric actin (*α*‐SA) (red). Nuclei were stained with DAPI (blue). Scale bar, 100 µm. i) Quantification results of PT‐BsAbs in the MI heart 6 h after inhalation or i.v. injection. *N* = 6.

MI was created by permanent ligation of the left anterior descending (LAD) in mice. PT‐BsAbs or NT‐BsAbs (CD34‐IgG) were pre‐labeled (DyLight 633) before inhalation by the MI mice, which were then observed with the IVIS imager. MI mice were randomized into three groups:1) inhalation of PT‐BsAbs, 2) inhalation of NT‐BsAbs, and 3) i.v. injection of PT‐BsAbs. The inhalation schematic is shown in **Figure** **2**a. To further investigate the heart homing ability, the forth group of mice was subjected to a sham surgery without LAD ligation, followed by inhalation of PT‐BsAbs. The inhalation delivery equipment information and protocol have been previously published.^[^ ^22^ ^]^ The BsAb solution was aerosolized for mice inhalation. Using the IVIS imager, the mean fluorescent intensity was measured at different time points as a positive‐linear reflection of their accumulation (Figure 2b). Inhalation delivery resulted in a faster and higher accumulation of PT‐BsAbs in the lungs compared to that from i.v. injection (Figure 2b). In addition, in MI mice, PT‐BsAbs were present in the heart 0.5 h after inhalation, and the accumulation further increased at 6 h (Figure 2b,c). The increased accumulation in the heart was accompanied by a decay in the lungs (Figure 2c), suggesting the transportation of PT‐BsAbs from the lungs to the heart. The retention time of PT‐BsAbs in the heart persisted up to 60 h (Figure 2b). In contrast, fewer PT‐BsAbs were transferred to the heart by i.v. injection. Both of the NT‐BsAbs in MI mice and PT‐BsAbs in sham mice only resulted in a negligible amount of antibodies in the heart (Figure 2b,d). It is notable that inhalation in MI mice also decreased off‐target loss of PT‐BsAbs to other major organs such as the kidneys, as compared to that of i.v. injection (Figure S5, Supporting Information). Such cardiac affinity was not seen in other groups. Immunofluorescence (IF) further confirmed the presence and binding of PT‐BsAbs to HSCs in the lungs. We carefully traced the route of PT‐BsAbs in MI mice after inhalation. 1 h after inhalation, a large quantity of PT‐BsAbs were presented in the lung parenchyma, indicating that they could penetrate the respiratory tract barrier, especially the bronchi (**Figure** **3**a,b). 6 h later (Figure 3c,d), lung accumulation of PT‐BsAbs reduced to about 35.4% of that at 1 h (Figure 3e), consistent with IVIS results mentioned above (Figure 2c,d). 81.9% of the PT‐BsAb signals at 6 h were merged with the signals for vessels in the lungs (Figure 3f), demonstrating the potential of PT‐BsAbs to transfer through the alveolar membrane to the pulmonary blood circulation. We then found that PT‐BsAbs or NT‐BsAbs mainly accumulated in the border zone of the MI heart, and we checked their accumulation in four groups 6 h after administration (Figure 3g). In MI mice, inhalation of PT‐BsAbs yielded an accumulation rate 3.4‐fold higher than that achieved from i.v. injection (Figure 3h). Fewer NT‐BsAbs went to the heart (Figure S6a, Supporting Information). As for the sham mice, even with the PT‐BsAbs inhalation, accumulation in the heart is negligible (Figure S7b, Supporting Information).

### 2.6 Inhaled Platelet‐Targeting Bispecific Antibody Redirects Lung Hematopoietic Stem Cells to the Injured Heart

**Figure 4:**
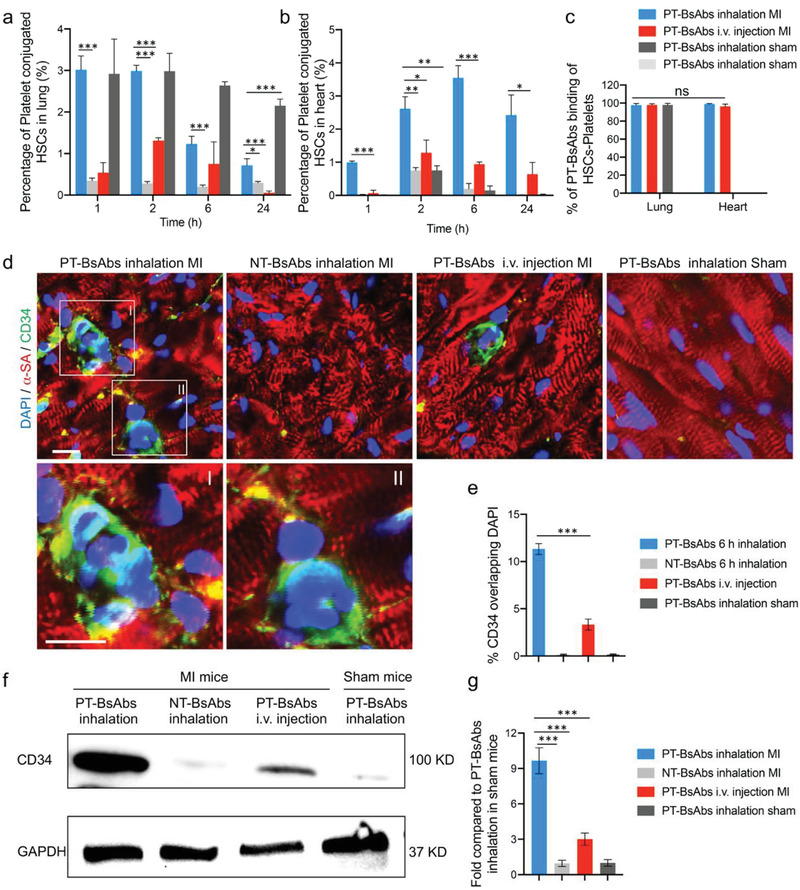
PT‐BsAb redirects HSCs to the injured heart. Flow cytometry quantification results of platelet‐conjugated HSCs (CD34^+^CD42b^+^) in a) the lungs and b) the heart. c) Flow cytometry quantification results of PT‐BsAbs binding of HSCs‐platelets (CD34^+^CD42b^+^DyLight 633^+^). HSCs and platelets were separately stained with CD34 antibody and CD42b antibody, while PT‐BsAbs were pre‐labeled with DyLight 633. d) Representative confocal images showing the accumulation of HSCs after the inhalation of PT‐BsAbs or NT‐BsAbs, i.v. injection of PT‐BsAbs in the border zone of MI mice, and inhalation of PT‐BsAbs in sham mice at 6 h. Cardiomyocytes were stained with *α*‐SA (red), while HSCs were stained with CD34 antibody (green). Nuclei were stained with DAPI (blue). Scale bar, 10 µm. e) Quantification of HSC accumulation in the heart 6 h after various administrations. f,g) Western blot analysis of CD34 expression in the heart 6 h after various treatments. *N* = 3.

PT‐BsAbs or NT‐BsAbs were pre‐labeled (DyLight 633) before administration. Mice with MI or sham surgery were randomized into four groups: 1) inhalation of PT‐BsAbs in MI mice, 2) inhalation of NT‐BsAbs in MI mice, 3) i.v. injection of PT‐BsAbs in MI mice, and 4) inhalation of PT‐BsAbs in sham mice. To investigate whether inhaled or injected BsAbs facilitated conjugation between HSCs and platelets, flow cytometry of lung and heart tissues was conducted at different time points. The percentage of platelet‐conjugated HSCs in the lung was shown in Figure S7a, Supporting Information, and **Figure** **4**a. These results showed that the inhalation of PT‐BsAbs in both MI mice and sham mice resulted in the high conjugation of HSCs with platelets within 2 h in the lung. However, the signal reduced in MI mice after 6 h, while it remained high in sham mice (Figure 4a). In the three groups of treated MI mice, inhalation of PT‐BsAbs caused a higher percentage of platelet‐conjugated HSCs compared to i.v. injection, which was consistent with the accumulation of PT‐BsAbs in the lungs in Figure 2. Inhalation of NT‐BsAbs resulted in less conjugation of platelets and HSCs due to the antibody's lack of platelet ligands. The DyLight 633 signal, labeling PT‐BsAbs, was detected in platelet‐conjugated HSCs. The quantified results, shown in Figure 4c, indicated that these cells were positive for DyLight 633, revealing that HSCs conjugated with the platelets as a result of the PT‐BsAbs. Immunofluorescence further confirmed the conjugation of PT‐BsAbs to HSCs in the lungs (Figure S8, Supporting Information). Flow cytometry results of platelet‐conjugated HSCs in the heart were shown in Figure S7b, Supporting Information, and Figure 4b. In sham mice, fewer HSCs were detected in the healthy heart, mainly because platelets were attracted to the injured heart, not the healthy heart. Inhalation of PT‐BsAbs in MI mice led to a higher percentage of platelet‐conjugated HSCs compared to other groups (3.8‐fold higher than i.v. injection of PT‐BsAbs, 17.7‐fold higher than inhaled NT‐BsAbs, and 23.6‐fold higher than inhaled PT‐BsAbs in sham mice 6 h post administration). Inhalation of PT‐BsAbs led to an accumulation of HSCs in the heart in a time‐dependent manner, accompanied with a signal decay in the lung, which was consistent with the translocation of PT‐BsAbs from the lungs to the heart (Figure 2). Notably, platelet‐conjugated HSCs in the heart were also Dylight 633 positive (Figure 4c), indicating that PT‐BsAbs mediated translocation of HSCs from the lungs. A dose‐escalation study was conducted to examine the accumulation of platelet‐conjugated HSCs in the MI heart after PT‐BsAb inhalation. The result showed the beneficial effect of 0.5 mg kg^−1^ of PT‐BsAbs in HSC translocation (Figure S10, Supporting Information). To further reveal the phenotypes of the recruited CD34^+^ cells in the heart, we used flow cytometry to examine surface markers including c‐kit, CD48, and CD150. The results indicated those CD34^+^ cells were c‐kit^+^Lin^−^CD150^+^CD48^−^ (Figure S9, Supporting Information), which was consistent with previous reported HSC phenotypes.^[^ ^23^ ^]^ The accumulation of HSCs and their morphology were further confirmed with immunofluorescence (Figure 4d,e) and western blotting (Figure 4f,g). These HSCs were located in the border zone of the MI heart and were close to cardiomyocytes. HSCs did not exist in other sites, including healthy sites (regions remote from the MI) and absolute MI areas (Figure S11, Supporting Information). Again, inhalation of PT‐BsAbs outperformed i.v. injection in redirecting HSCs to the heart. In contrast, negligible signals of HSCs were observed in NT‐BsAbs MI group and PT‐BsAbs sham group.

### 2.7 Platelet‐Targeting Bispecific Antibody Inhalation Therapy Reduces Apoptosis and Modulates Inflammation

**Figure 5:**
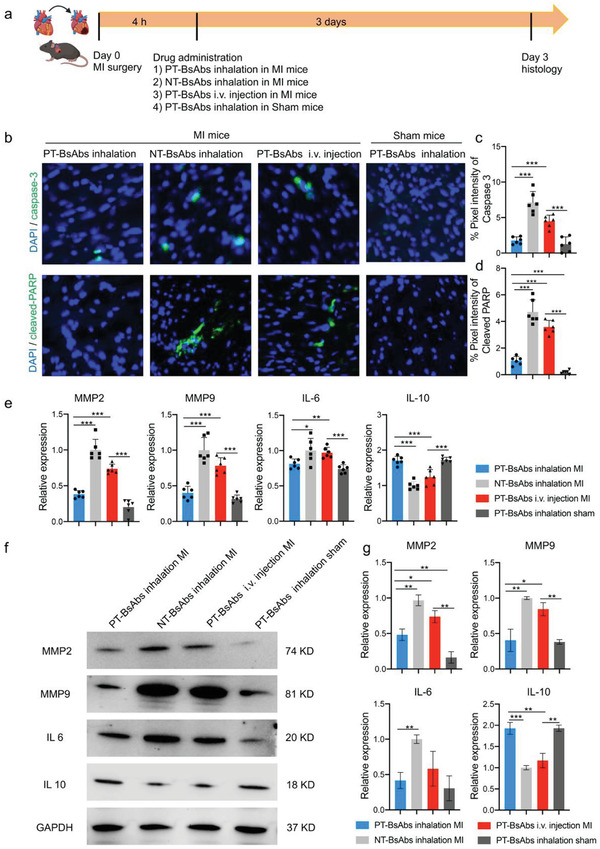
Inhalation of PT‐BsAbs promotes short‐term cardiac repair post MI. a) Schematic illustrating the overall design of animal experiments to test the short‐term therapy efficiency of PT‐BsAbs in the border zone of the MI mice. b) Representative Caspase‐3 and cleaved PARP immunostaining of apoptotic cells in the border zone of MI mice and sham mice 3 days post different treatments. Apoptotic cells were stained in green, and nuclei were stained with DAPI (blue). Scale Bar, 50 µm. Quantification of percentage of c) Caspase‐3 and d) cleaved PARP. *N* = 6. e) Inflammation‐related mRNA expression in the heart 3 days post treatments. *N* = 6. f) Inflammation‐related protein expressions in the heart 3 days post treatments and g) quantification. *N* = 3.

After showing that the PT‐BsAbs redirected HSCs to the injured heart, we investigated the effects of that redirection on cardiac repair. MI mice or sham mice were randomized into four groups: 1) inhalation of PT‐BsAbs in MI mice, 2) inhalation of NT‐BsAbs in MI mice, 3) i.v. injection of PT‐BsAbs in MI mice, and 4) inhalation of PT‐BsAbs in sham mice (schematic in **Figure** **5**a). After 3 days, in the border zone of MI mouse hearts, PT‐BsAbs inhalation led to a reduction in the early stage of apoptosis (Figure 5b–d) (*p* < 0.001 for caspase‐3 and cleaved PARP, respectively). We further investigated the anti‐inflammatory ability of the inhaled PT‐BsAbs in MI mice (Figure 5e). There was a reduction in inflammation‐related mRNAs, such as MMP2 and MMP9 after treatment. In addition, as a pro‐inflammatory factor, interleukin‐6 (IL‐6) plays an essential role in accelerating the inflammation process.^[^ ^24^ ^]^ Expression levels of IL6 were decreased after PT‐BsAbs inhalation in MI mice. Interestingly, PT‐BsAbs treatment increased IL‐10 expression, which is an important indicator of anti‐inflammation.^[^ ^25^ ^]^ Inflammation‐related protein expressions were further evaluated and quantified (Figure 5f,g), showing consistent trends with mRNA expression levels. Collectively, those results demonstrated that PT‐BsAbs inhalation facilitated the formation of a post‐MI anti‐inflammation microenvironment, which was beneficial for cardiac repair.

### 2.8 Platelet‐Targeting Bispecific Antibodies Therapy Leads to Reparative Macrophage Polarization

**Figure 6:**
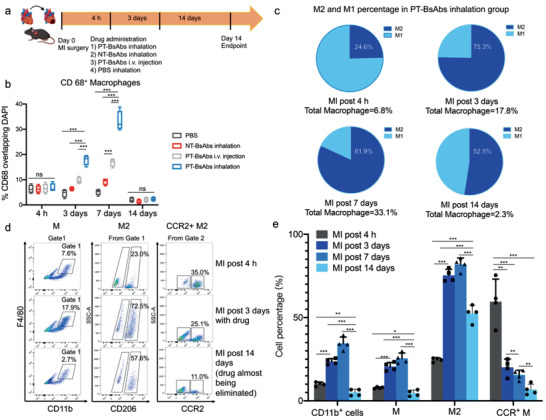
PT‐BsAb inhalation modulates macrophage subtypes in the injured heart. a) Schematic illustrating the overall design of animal experiments to test the effect on macrophages of different treatments. b) Quantification of CD68^+^ macrophages after different treatments at 4 h (before treatments), 3 days, 7 days, and 14 days (with treatments). The result of NT‐BsAbs group was not shown. c) Quantification of M1‐like and M2‐like macrophages in the heart of mice treated with PT‐BsAbs via inhalation administration. d) Flow cytometry results of macrophage percentage (M) were shown in gate 1. Macrophages were stained with CD11b^+^F4/80^+^. M2‐like macrophages were stained with CD11b^+^F4/80^+^CD206^+^ and CCR2^+^ macrophages was stained with CD11b^+^F4/80^+^CCR2^+^. e) Quantitative analysis of results from figure (d) in different time points. *N* = 4.

The PT‐BsAbs’ ability to create an anti‐inflammation microenvironment, especially their enhancement of IL‐10, inspired us to investigate the polarization of macrophages in the post‐MI microenvironment. MI mice were randomized into the following four treatment groups: 1) PBS inhalation, 2) NT‐BsAbs inhalation, 3) i.v. injection of PT‐BsAbs, and 4) PT‐BsAbs inhalation. Mice were sacrificed, and cardiac macrophages were analyzed at different timepoints: 4 h (before treatments) and 3,7, and 14 days post MI (**Figure** **6**a). CD68^+^ macrophages accounted for about 6–7% of total cells in the MI area for each group before the treatments (Figure S12, Supporting Information, Figure 6b). PT‐BsAbs inhalation led to an increase of those cells (17.8% at day 3 and 33.1% at day 7). In contrast, intravenously injected PT‐BsAbs also induced an increase in CD68^+^ macrophages, with a peak of 16.7% at day 7. Other treatment groups did not increase the number of CD68^+^ macrophages. 14 days later, CD68^+^ macrophages were reduced, and the percentages in all groups were even less than that 4 h post MI, which was consistent with the published literature.^[^ ^26^ ^]^ We then delineated the subtypes of macrophages, including iNOS^+^ M1‐like macrophages and CD206^+^ M2‐like macrophages (Figure S13, Supporting Information). The ratio between the two in the inhaled PT‐BsAbs groups was quantified (Figure 6c). In the early stage of MI (4 h later), M1‐like macrophages were the most abundant, with negligible M2‐like macrophages. Without effective accumulation of PT‐BsAbs, the percentage of M1‐like macrophages increased after 3 days (PBS group). This tide was reversed by PT‐BsAbs treatment, which led to a high ratio of M2‐like macrophages (3.0‐fold higher than that of M1‐like macrophages) (Figure 6d) after 3 days. These results were further verified by flow cytometry (Figure 6d,e, Figure S14), revealing the increased percentage of M2‐like macrophages after PT‐BsAbs administration. In addition, inhaled PT‐BsAbs resulted in a reduction of CCR2^+^ (C‐C chemokine receptor 2) macrophages (20.0% at day 3 vs 59.5% 4 h post MI, *p* < 0.01). It has been reported that neutrophils also play an essential role in ischemic cardiac repair.^[^ ^27^, ^28^ ^]^ We examined neutrophils at day 7 post MI,^[^ ^29^ ^]^ but we did not find a significant difference after PT‐BsAb administration as compared to other groups in the MI heart (Figure S15, Supporting Information).

### 2.9 Platelet‐Targeting Bispecific Antibody Inhalation Therapy Boots Cardiac Function and Attenuates Cardiac Remodeling

**Figure 7:**
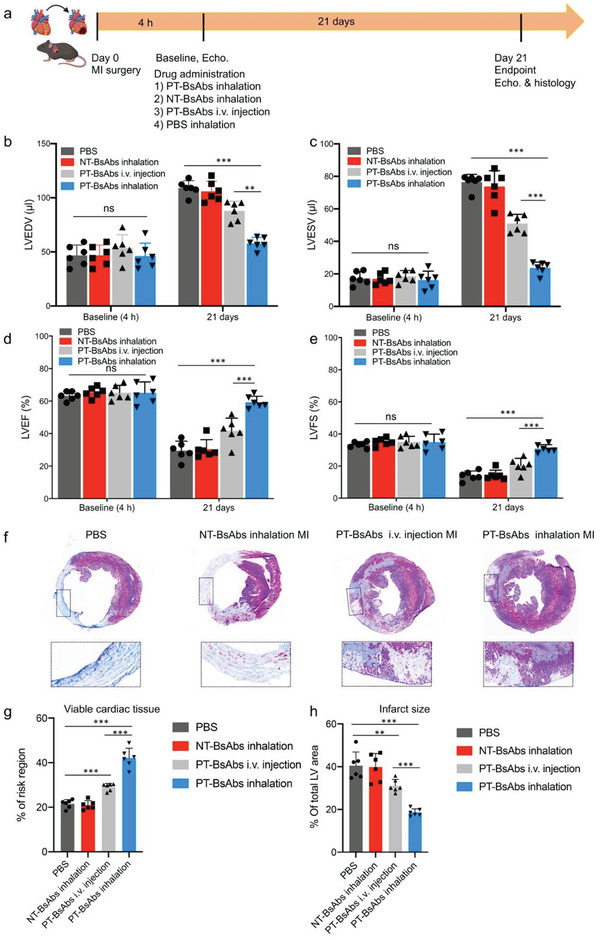
PT‐BsAb inhalation improves cardiac function and promotes cardiac repair post MI. a) Schematic illustrating the overall design of animal experiments to test the therapy efficiency of PT‐BsAbs 21 days after treatments in MI mice. b) LVEDV, c) LVESV, d) LVEF, and e) LVFS measured by echocardiography at baseline (4 h post MI) and 21 days afterward in control PBS, control inhalant NT‐BsAbs, i.v. injection of PT‐BsAbs, and PT‐BsAbs with inhalation administration groups. f) Masson's trichrome images showing scar (blue) and viable (red) tissues in the heart at 21 days post treatments. Morphometric parameters including the percentage of g) viable cardiac tissues and h) infarct size measured from Masson's trichrome images via NIH Image J software. *N* = 6.

To investigate the therapeutic effect of PT‐BsAbs via inhalation, MI mice were randomly assigned to four groups: 1) PBS, 2) inhalation of NT‐BsAbs, 3) i.v. injection of PT‐BsAbs, and 4) inhalation of PT‐BsAbs. The different treatments were administrated 24 h post MI (Schematic in **Figure** **7**a). The cardiac function indicators 4 h post MI were measured as baselines, and also monitored for 21 days after treatments before sacrificing mice atendpoint (Supporting information Movies S1–S4). Echocardiography quantitative results demonstrated decreased left ventricular (LV) end‐diastolic volume (LVEDV) and LV end‐systolic volume (LVESV), and enhanced LV ejection fractions (LVEF) and LV fractional shortening (LVFS) values after the treatment of inhalant PT‐BsAbs, compared to other groups (Figure 7b–e). It indicated the attenuation of cardiac remodeling due to PT‐BsAbs treatment. Specifically, mice treated with i.v. injection of PT‐BsAbs were only 70.2% and 66.1% (respectively) of the LVEF and LVFS values of those that received inhalation therapy (*p* < 0.001). The i.v. injection of PT‐BsAbs group showed an slight increase in these figures compared to the PBS and NT‐BsAbs groups. Cardiomyocytes have been shown to proliferate in vivo,^[^ ^30^ ^]^ albeit rarely. This ability can be damaged under ischemic conditions. Masson's trichrome‐staining of the hearts (Figure 7f) indicated the increase in viable cardiac area (Figure 7g) and attenuated infarction size (Figure 7h) after the administration of the inhaled PT‐BsAbs.

### 2.10 Platelet‐Targeting Bispecific Antibody Inhalation Therapy Modulates the Post‐Myocardial Infarction Microenvironment

**Figure 8:**
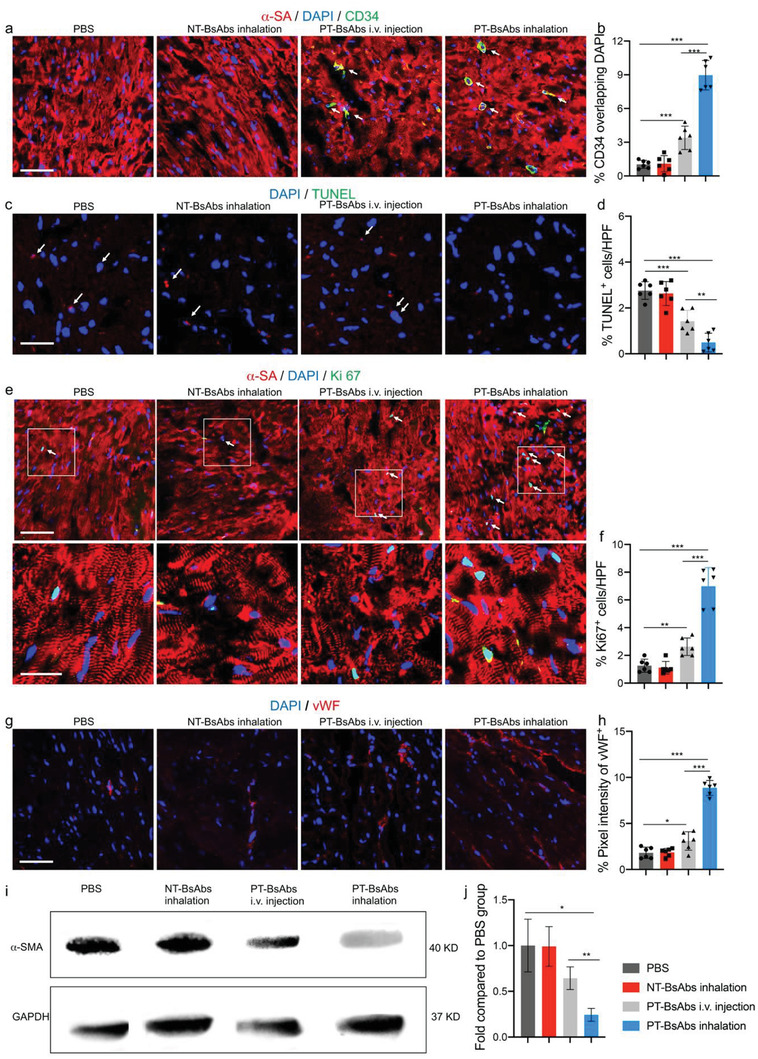
PT‐BsAb inhalation promotes angiomyogenesis and reduces cell death in the border zone of the MI heart. a) Confocal microscopic images and b) quantification confirming that inhaled PT‐BsAbs promoted HSC accumulation 21 days post the treatment (*n* = 6). Cardiomyocytes were stained with *α*‐SA antibody (red), and HSCs were stained with CD34 antibody (green). Nuclei were stained with DAPI (blue). Scale bar, 100 µm. c) Representative TUNEL immunostaining and d) quantification of apoptotic cells in each treatment group (*n* = 6). Scale bar, 200 µm. White arrowheads indicated TUNEL‐positive cells. e) Confocal microscopic images showing Ki67‐positive cardiomyocytes and nuclei (blue) in each treatment group (*n* = 6). Scale bar, 50 and 25 µm for higher magnification snapshot. White arrowheads indicated Ki67‐positive cardiomyocytes. f) The numbers of Ki67‐positive nuclei were quantified. g) Confocal microscopic images showing vWF‐positive signal and nuclei (blue) in each treatment group (*n* = 6). Scale bar, 100 µm. h) The numbers of vWF‐positive nuclei were quantified. i) Western blot analysis of *α*‐SMA expression in each treatment group. GAPDH was used as a control. j) The normalized *α*‐SMA expression levels were quantified (*n* = 3).

Following 21 days of treatments (Figure 7a), inhalation therapy of PT‐BsAbs led to a therapeutic homing of HSCs to the border zone of the MI hearts (**Figure** **8**a,b). Those HSCs were surrounded by cardiomyocytes. PT‐BsAb inhalation also led to a reduction in apoptosis (Figure 8c,d) (*p* < 0.001). Ki 67 is an indicator of proliferation and a cell cycling marker. It is especially expressed during the G1, S, and G2 stages, and even during early mitosis.^[^ ^31^ ^]^ The expression percentage of Ki 67 for the groups that received the PT‐BsAbs inhalation therapy was 2.7, 6.3, and 6.2‐fold higher than that of the injected PT‐BsAbs (*p* < 0.001), NT‐BsAbs, and PBS groups (*p* < 0.001), respectively (Figure 8e,f). vWF is used to evaluate the presence of microvascular angiogenesis. Our results demonstrate an increase in capillary density in the border zone of the infarct area after inhaled PT‐BsAbs treatment, and the pixel intensity of vWF^+^ was about 2.9‐fold of that in MI mice treated with i.v. injection of PT‐BsAbs and about 4.9‐fold of that in MI mice treated with PBS (*p* < 0.001 for both) (Figure 8g,h). On the other hand, myofibroblasts respond to MI induced fibrosis, accompanied by high expressions of *α*‐smooth muscle actin (*α*‐SMA). Fibrosis plays a key role in remodeling, but excessive fibrosis can lead to heart failure. We evaluated *α*‐SMA expression through western blotting (Figure 8i,j). Similarly to other MI therapies,^[^ ^32^ ^]^ inhaled PT‐BsAbs dramatically reduced the expression level of *α*‐SMA. These consequences revealed the benefits of PT‐BsAbs in promoting angiogenesis and reducing fibrosis in the post‐MI heart. These results also highlighted the importance of myofibroblast targeting for MI therapy.^[^ ^33^ ^]^

### 2.11 Platelet‐Targeting Bispecific Antibody Inhalation Therapy Does not Cause Toxicity

The biocompatibility and safety of PT‐BsAbs were investigated. H&E staining of the main organs (including liver, spleen, lung, kidneys) did not show histological abnormity after therapies (Figure S16, Supporting Information), suggesting negligible systemic toxicity. Negligible CD68^+^ macrophages, CD8^+^ T cells, and CD4^+^ T cells were found in the heart (Figure S17, Supporting Information) and in the lung (Figure S18, Supporting Information) among all groups.

## 3 Discussion

Various approaches such as magnetic targeting and antibody targeting have been employed to address the low cardiac retention rate after cell injection.^[^ ^34^, ^35^ ^]^ Relying on the natural homing property of platelets, we constructed PT‐BsAbs to conjoin HSCs with platelets and redirect lung‐based HSCs to the injured heart. Click chemistry was used for the synthesis of PT‐BsAbs. Previous researchers have used intravenously injected antibodies to recruit stem cells released from bone marrow and bring these cells to the cardiac injury site.^[^ ^11^ ^]^ However, it is notable that HSCs only account for 3% of cells in the bone marrow and the number is even more sparse in the circulation.^[^ ^21^ ^]^ It was recently reported that the lung is a reservoir for HSCs and platelets.^[^ ^17^ ^]^ This inspired us to design a “from the lung to the heart” strategy: nebulize PT‐BsAbs to the lungs to capture HSCs (Figure 1a) and bridge them with platelets (Figure 4a,c) in the lungs. With the platelet's natural homing property, PT‐BsAbs can redirect HSCs via pulmonary blood circulation (Figure 3c,d) to the heart (Figure 4b,d). Given the abundance of HSCs and platelets in the lung, this could be the reason why PT‐BsAbs inhalation had more therapeutic benefits than i.v. injection of PT‐BsAbs (Figure 4). In addition, the advantage of inhalation could be explained by the heart being the first “capillary narrowing” after the lung. It means that platelet‐conjugated HSCs in the lung are easier to go to the heart. Those PT‐BsAbs coming into the circulation could further conjoin platelets and HSCs followed by infiltrating to the heart. In comparison, if PT‐BsAbs were i.v. injected, then the lung would be the first area where these conjugations would be stuck. In addition, the inhalation route has been well established as a safe and effective approach for pulmonary drug delivery.^[^ ^36^, ^37^ ^]^

The use of platelets such as platelet mimics or antibody conjugated platelets have been considered as versatile platforms for drug delivery and regenerative medicine therapies.^[^ ^38^, ^39^ ^]^ However, some issues need to be addressed before further translation. Platelets have been reported to be the first wave of cells infiltrated in the myocardium after ischemic/reperfusion (I/R) injury, and myocardial ischemia may also directly activate platelets.^[^ ^40^, ^41^ ^]^ Activated platelets infiltrated into ischemic myocardium contribute to injury deterioration by the formation of microthrombi and the release of pro‐inflammatory molecules.^[^ ^40^ ^]^ Notably, only activated platelets act as culprits in accelerating injury, and platelet activation has not been found in sham‐operated animals.^[^ ^41^ ^]^ Therefore, deactivated platelets and their mimics have been widely developed especially in MI therapy, showing dramatically heart accumulation.^[^ ^13^, ^14^ ^]^ Overall, platelet based platform technique shows promising potential in injury homing due to their multiple surface receptors.^[^ ^12^ ^]^ Interestingly, bone marrow MSC‐platelet aggregates have been found in patients with MI than in healthy control, which contributed to better prognosis.^[^ ^42^ ^]^

Our strategy has several innovation points. First, BsAb therapy is mainly used for treating cancers and autoimmune diseases.^[^ ^43^, ^44^ ^]^ We reported a PT‐BsAb to treat cardiovascular disease. Second, we leveraged the lung's biogenesis to repair the heart. Third, we used inhalation delivery instead of intravenous injection. Inhalation of PT‐BsAb was more efficient and specific than the intravenous route (Figure 2, five‐fold enhancement at 2 h). Moreover, G‐CSF facilitates the release of stem cells from the bone marrow into peripheral blood circulation.^[^ ^45^ ^]^ However, the SITAGRAMI trail failed to show a beneficial effect on cardiac function.^[^ ^46^, ^47^ ^]^ The PT‐BsAb strategy is designed to address the targeting issue of released stem cells. We also explored the mechanisms underlying the therapeutic benefits of PT‐BsAb therapy. HSCs have limited potential to transdifferentiate into cardiomyocytes.^[^ ^48^ ^]^ Our data suggested that the accumulated HSCs, in a short period of time, improved cardiac repair and modulated inflammation in the MI microenvironment (Figure 5). Furthermore, the redirected HSCs modified the phenotypes of macrophages, favoring the anti‐inflammation and pro‐regeneration M2 macrophages (Figure 6c). It has been reported that CCR2 signaling could overexpress after MI, recruiting monocytes and educating them into CCR2^+^ macrophages.^[^ ^49^ ^]^ CCR2^+^ macrophages initiated inflammation, and the inhibition of CCR2^+^ was developed as a new strategy for MI treatment.^[^ ^50^, ^51^ ^]^ Interestingly, in our results, inhaled PT‐BsAbs dramatically reduced the percentage of CCR2^+^ macrophages (Figure 6d,e). On top of that, PT‐BsAb treatment also let to an increase in angiomyogenesis and decrease in apoptosis in the post‐MI heart (Figure 8).

Our strategy also has a few of limitations. First, the pathological conditions in the lung may affect the ability of the lung to produce HSCs and platelets. In addition, the extravasation mechanism of PT‐BsAbs has not been understood clearly. Furthermore, the Fc domains of whole antibodies may encounter side‐effects of the immunogenicity even though the PT‐BsAbs used in our experiments indicated safety. Future studies may use Fab fragments or ScFv to replace whole antibody versions of the PT‐BsAbs. Moreover, further toxicity studies using a much higher dose (e.g., tenfold of the therapeutic dose) is needed before clinical translation.

## 4 Experimental Section

##### Mouse Model of Myocardial Infarction

Male C57BL/6 mice were purchased from Charles River Laboratories (8–10 week old). All animal work was compliant with the Institutional Animal Care and Use Committee (IACUC) at University of North Carolina at Chapel Hill and North Carolina State University. MI models were induced as previously described.^[^ ^52^ ^]^ Mice were anesthetized by i.p. injection of K‐X cocktail (ketamine: 100 mg kg^−1^, xylazine: 10 mg kg^−1^ body weight), while artificial ventilation (SAR‐1000 Small Animal Ventilator, CWE, Inc.) provided life support for these mice. Under sterile condition, the ischemia was achieved by permanently ligating the LAD coronary artery.

##### Construction of Platelet‐Targeting Bispecific Antibodies

Rat anti‐mouse CD34 and Mouse anti‐CD42b antibodies were initially reacted with TCO‐PEG‐NHS and TZ‐PEG‐NHS by acylation reaction between ‐NHS and ‐NH_2_, respectively. In detail, CD34 (1–5 mg mL^−1^) and TCO‐PEG‐NHS or CD42b (1–5 mg mL^−1^) and TZ‐PEG‐NHS were mixed and reacted at 4 °C for 24 h. Unreacted TCO‐PEG‐NHS and TZ‐PEG‐NHS were removed via dialysis (Slide‐A‐Lyzer MINI Dialysis Units, 10 000 MWCO) and centrifugation (Amicon Ultra‐0.5 filter, 100 kDa). IgG was reacted with TCO‐PEG‐NHS or TZ‐PEG‐NHS to synthesize IgG‐TCO and IgG‐TZ for the flowing usage. PT‐BsAbs were synthesized via a chemical click reaction. Equimolar of the acquired CD34‐TCO and CD42b‐TZ were mixed and reacted at 4 °C for 48 h via the linkage between TCO and TZ. Final BsAbs could be concentrated by the ultrafiltration method. Murine IgG‐IgG, CD42b‐IgG, and CD34‐IgG (NT‐BsAbs) were respectively synthesized with the same method as control groups.

##### Characterization of Platelet‐Targeting Bispecific Antibodies

The identify of CD34‐TCO, CD42b‐TZ, and PT‐BsAbs were confirmed by SDS‐PAGE. Semi‐products in control groups were also evaluated by SDS‐PAGE. Given the different species of CD34 (rat) and CD42b (mouse), transmission electron microscopy was employed to view PT‐BsAbs, labeled with goat anti‐rat gold nanoparticles (25 nm) and goat anti‐mouse gold nanoparticles (5 nm).

##### Binding Efficiency of Platelet‐Targeting Bispecific Antibodies to Hematopoietic Stem Cells or Platelets

Murine platelets were isolated from mouse blood as previously described.^[^ ^13^ ^]^ Collected platelets (total of 1 × 10^6^) were incubated with 2 µg of PT‐BsAbs at 37 °C for 4 h, followed by centrifugation at 600 × *g* for 8 min to discard unbound PT‐BsAbs. DiI‐labeled platelets were then incubated with FITC‐labeled goat anti‐rat CD34 antibody for 1 h, before being fixed and observed with a confocal microscope (Philip FLUOVIEW). HSCs were isolated from the lung and the bone marrow via isolation kit (BD) and sorted by flow cytometry (LSR II, BD). The content of HSCs from different tissues was analyzed, and CD34^+^ HSCs in the lungs were used for the following experiments. 1 × 10^4^ CD34^+^ HSCs were seeded on 4‐well plate followed by DiI staining, then 2 µg of PT‐BsAbs were added for 4 h. Cells were washed three times with PBS to remove dissociative PT‐BsAbs. After, FITC‐labeled goat anti‐mouse CD42b antibody were added and incubated with HSCs for another 1 h. Finally, cells were washed three times with PBS and stained with DAPI for 10 min. Confocal microscopy was used to image HSCs. HSCs incubated with CD42b‐IgG were also conducted as a control group.

##### Conjoining Ability of Platelet‐Targeting Bispecific Antibodies

DiO‐labeled HSCs were incubated with PT‐BsAbs for 4 h, followed by incubation with DiI labeled platelets. After 4 h, HSCs were washed and fixed with DAPI before being observed with confocal microscope. HSCs incubated with platelets in the existence of IgG‐IgG were also conducted as a control group. The conjugation was further confirmed by flow cytometry. HSCs were first incubated with pre‐labeled PT‐BsAbs (Novus Biological Lightning‐Link Rapid DyLight 633 Antibody Labeling Kit). Then DiO labeled platelets were added to incubate for another 4 h. Finally, cells were then collected and analyzed with flow cytometry (LSR II, BD).

##### Assessment of Biodistribution and the Short‐Term Study

PT‐BsAbs and NT‐BsAbs were pre‐labeled (Novus Biological Lightning‐Link Rapid DyLight 633 Antibody Labeling Kit) before use. Mice were divided into four groups (*n* = 3–6 for each group). Three groups were MI mice, which were given inhalation of PT‐BsAbs, inhalation of NT‐BsAbs, and i.v. injection of PT‐BsAbs, respectively (0.5 mg kg^−1^ body weight, dissolved in 0.3 mL PBS for each group). In addition, another group of mice was given sham surgery and administered inhaled PT‐BsAbs by the same dosage mentioned above. At each time point, mice were sacrificed and main tissues were imaged using the IVIS system. Fluorescent signal data were recorded by living image software version 3.0. For the short‐term study, mice were sacrificed 3 days after treatments followed by histology analysis.

##### Treatment Groups for the Long‐Term Therapeutic Study

MI mice were established and randomized into 4 groups (*n* = 6 for each group): 1) inhalation of PBS, 2) inhalation of NT‐BsAbs, 3) i.v. injection of PT‐BsAbs, and 4) inhalation of PT‐BsAbs (0.5 mg kg^−1^ body weight, dissolved in 0.3 mL PBS for each group). After, MI mice were accepted different therapies as each group. A cohort of mice were sacrificed after 21 days for analysis.

##### Cardiac Function Assessment with Echocardiogram

Cardiac function was measured by a cardiologist blinded to group designation. Echocardiography was performed using a Philips CX30 ultrasound system equipped with an L15 high‐frequency probe and Prospect T1 ultrasound system with a 40 MHz probe. Prior to measurements, mice were anesthetized by inhalation of 1.5% isoflurane‐oxygen mixture in supine position 4 h and 21 days after LAD ligation. At a level of the greatest LV diameter, hearts were imaged 2D in long‐axis views. LVEF was calculated by measuring LVEDV and LVESV: LVEFs% = (LVEDV‐LVESV)x100/LVEDV. LVFS was calculated as well in each echocardiogram.

##### Western Blot of Mouse Hearts

Tissue samples were lysed with a Radio‐Immunoprecipitation Assay buffer and the extracted protein were quantified by BCA assay (Invitrogen, CA) following the manufacturer's protocols. The protein solution was mixed with 4 × buffer containing reducing reagent and heated at 95 °C for 5 min. Then, protein samples were separated by 4–12% SDS‐PAGE electrophoresis (Invitrogen), after which they were transferred to polyvinylidene difluoride (PVDF) membranes (Bio‐Rad). PVDF membranes containing proteins were blocked with 3–5% bovine serum albumin at room temperature for 1 h and then incubated with primary antibodies at 4 °C overnight. GAPDH was used as a control. After being washed and incubated with secondary antibodies, PVDF membranes were detected using ECL western blotting substrate (Thermo Fisher Scientific, IL).

##### Immunofluorescence and Histology

IF staining was conducted on frozen sections of heart and lung from MI mice models. Tissues were dehydrated with 10%, 20%, and 30% sucrose solution overnight before being snap frozen in O.C.T. (Tissue‐Tek). Cryo‐sections were fixed with 4% paraformaldehyde (PFA) for 30 min, followed by being permeabilization and blocking with protein block solution (DAKO) containing 0.1% saponin for 1 h. Slices were incubated with primary antibodies at 4 °C overnight, and incubated with secondary antibodies at room temperature for 1.5 h. Primary and secondary antibodies were shown in Table S1, Supporting Information. After the nucleus staining with 10 min submersion of DAPI, slices were mounted with Pro‐Long Gold mounting solution (Thermo Fisher Scientific). IF images were captured on a confocal microscope (Philip, FLUOVIEW). Quantitation analysis of positive signals was analyzed by ImageJ software. Masson's trichrome staining was performed according to manufacturer's protocols and images were captured by a PathScan Enabler IV slide scanner (Advanced Imaging Concepts, Princeton, NJ). Viable myocardium was quantified by ImageJ software.

##### Flow Cytometry

Single‐cell suspensions of heart samples from MI models were used to analyze macrophage with flow cytometry. Briefly, whole heart tissues were digested and grounded into single‐cell suspensions. After red blood cell lysis (AKT buffer), cells were diluted into 1 × 10^6^ cells mL^−1^ in PBS. 1 mL of cell suspension was centrifuged followed by incubation with primary antibodies or fluorescence conjugated antibodies at room temperature for 30 min. As for samples with unconjugated primary antibodies, cells were centrifuged and added by fluorescent secondary antibodies for another 30 min at room temperature. Cells were finally fixed with 4% PFA and analyzed with FACS (LSR II, BD). Unconjugated primary antibodies and fluorescent secondary antibodies or fluorescence conjugated antibodies were shown in Table S1, Supporting Information. Data were analyzed with FlowJo software (TreeStar, Ashland, OR).

##### qPCR

Total RNA was extracted from heart tissues with MI with a RNeasy Mini Kit (Qiagen). Then, cDNA was reverse‐transcribed with an iScript cDNA Synthesis Kit (BIO‐RAD), and 500 ng for each sample was used for amplification with TaqMan Gene Expression Master Mix. All primers for qPCR reactions were purchased from TaqMan, while GAPDH was used as the endogenous control. Reactions were performed by 7500 Real‐Time PCR System and data were analyzed with relevant 7500 software.

##### Data Analysis

Data were expressed as mean ± standard deviation (SD). Statistical significance were performed with a two‐tail unpaired Student's *t*‐test when comparing two groups. Comparisons among three groups or more were conducted with one‐way ANOVA, followed by the post hoc Bonferroni test. The probability value of *p* < 0.05 was considered significant, which were documented in figures. * indicated *p* < 0.05, ** indicated *p* < 0.01, *** indicated *p* < 0.001, respectively.

## Conflict of Interest

The authors declare no conflict of interest.

## Supporting information

Supporting Information

Supplemental Movie 1

Supplemental Movie 2

Supplemental Movie 3

Supplemental Movie 4
