# Supplementary material for: Bispecific Antibody Inhalation Therapy for Redirecting Stem Cells from the Lungs to Repair Heart Injury
Source: Adv Sci (Weinh). 2020 Nov 19;8(1):2002127. doi: 10.1002/advs.202002127 (PMC7788635; doi:10.1002/advs.202002127)
Supplement: Supplementary file 1 — Supporting Information [file ADVS-8-2002127-s001.pdf]

© 2020 Wiley-VCH GmbH

ADVANCED SCIENCE
Open Access

Supporting Information

for *Adv. Sci.,* DOI: 10.1002/advs.202002127

Bispecific antibody inhalation therapy for redirecting stem cells from the lungs to repair heart injury

Mengrui Liu[1,2], Halle Lutz[1], Dashuai Zhu[1,2], Ke Huang[1], Zhenhua Li[1,2], Phuong-Uyen C. Dinh[1,3], Junqing Gao[4], Yi Zhang[5], Ke Cheng[1,2] *

Supplementary Materials

Bispecific antibody inhalation therapy for redirecting stem cells from the lungs to repair heart injury

Mengrui Liu[1,2], Halle Lutz[1], Dashuai Zhu[1,2], Ke Huang[1], Zhenhua Li[1,2], Phuong-Uyen C. Dinh[1,3], Junqing Gao[4], Yi Zhang[5], Ke Cheng[1,2] *

[1] Department of Molecular Biomedical Sciences, North Carolina State University, North Carolina, USA.
[2] Joint Department of Biomedical Engineering, University of North Carolina at Chapel Hill and North Carolina State University, USA.
[3] Comparative Medicine Institute, North Carolina State University, North Carolina, USA.
[4] Department of Cardiology, Putuo Hospital, Shanghai University of Traditional Chinese Medicine, Shanghai, China.
[5] Department of Cardiology, Shanghai Tenth People's Hospital, Tongji University School of Medicine, Shanghai, China.
* Corresponding Author: Ke Cheng, Ph.D., North Carolina State University, 1001 William Moore Dr, Raleigh, NC 27606, Email: kcheng3@ncsu.edu

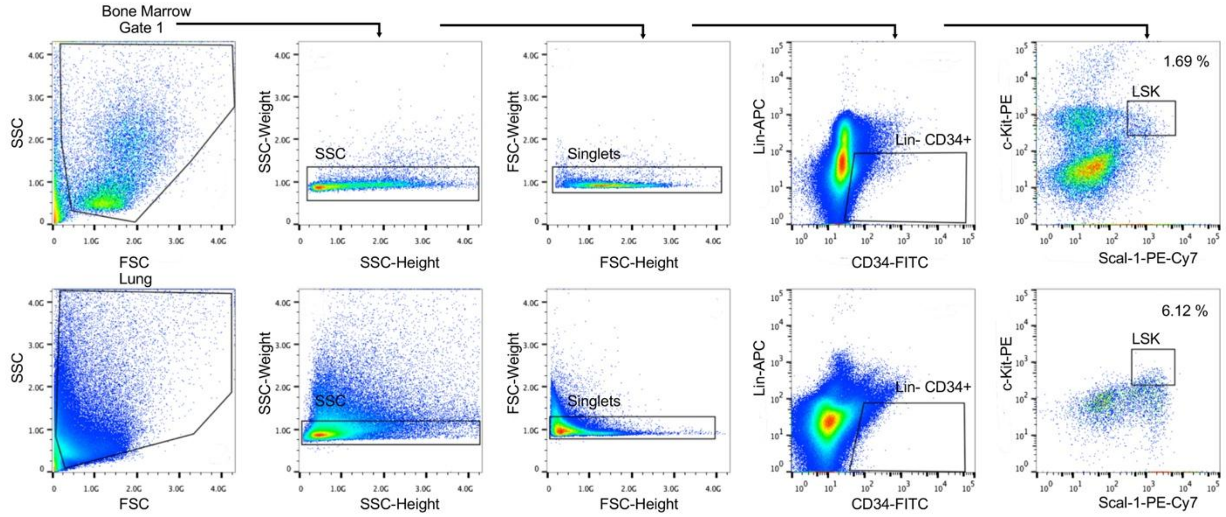

Figure S1. The lung contains a significant amount of HSCs. Representative flow cytometry dot plots showing the percentage of HSCs in the bone marrow and in the lungs. HSCs were identified as $Lin^{-}Sca\text{-}1^{+}c\text{-}Kit^{+}CD34^{+}$.

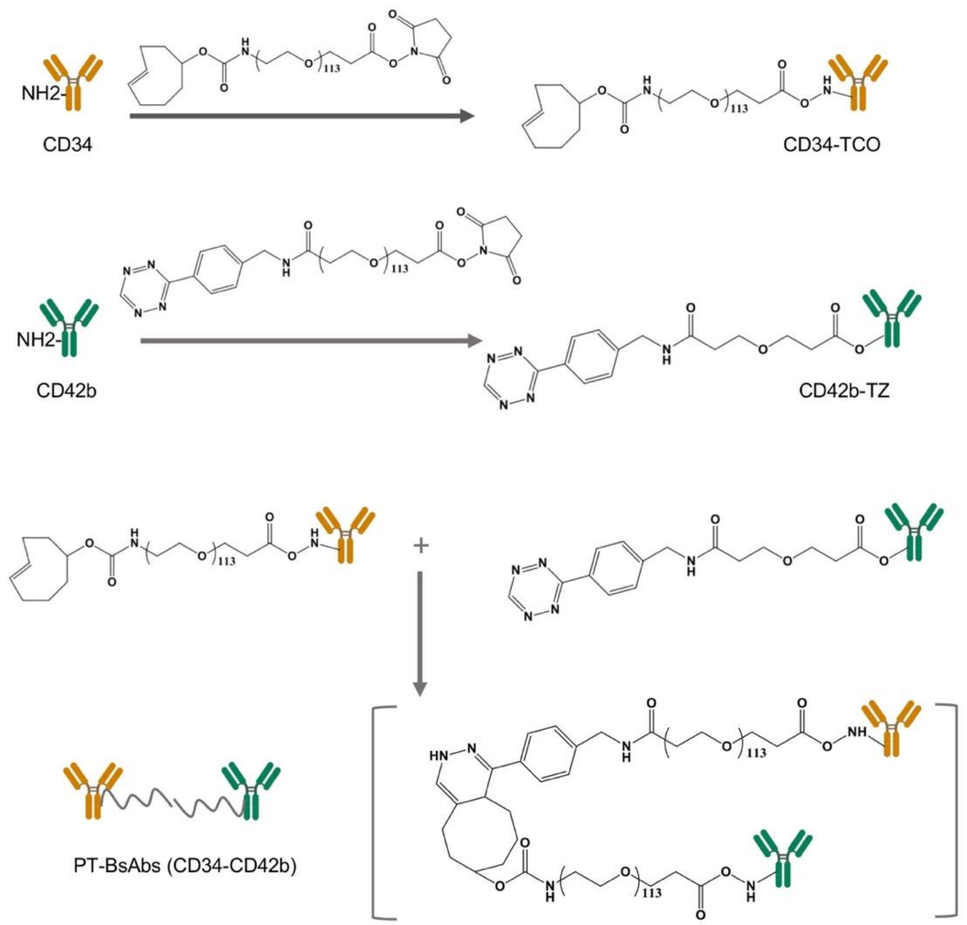

Figure S2. Synthesis process of CD34-TCO, CD42b-TZ and PT-BsAbs (CD34-CD42b).

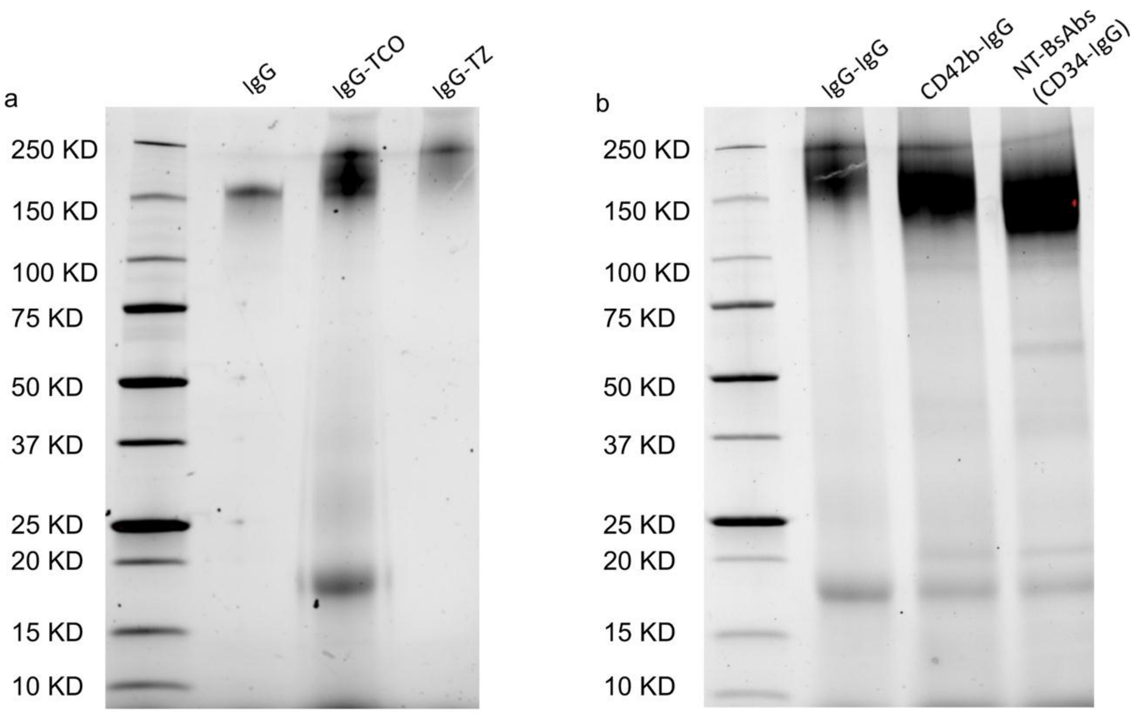

Figure S3. SDS-PAGE results. (a) SDS-PAGE of IgG and semi-products (IgG-TCO and IgG-TZ). (b) SDS-PAGE of synthesized IgG-IgG, CD42b-IgG and NT-BsAbs (CD34-IgG).

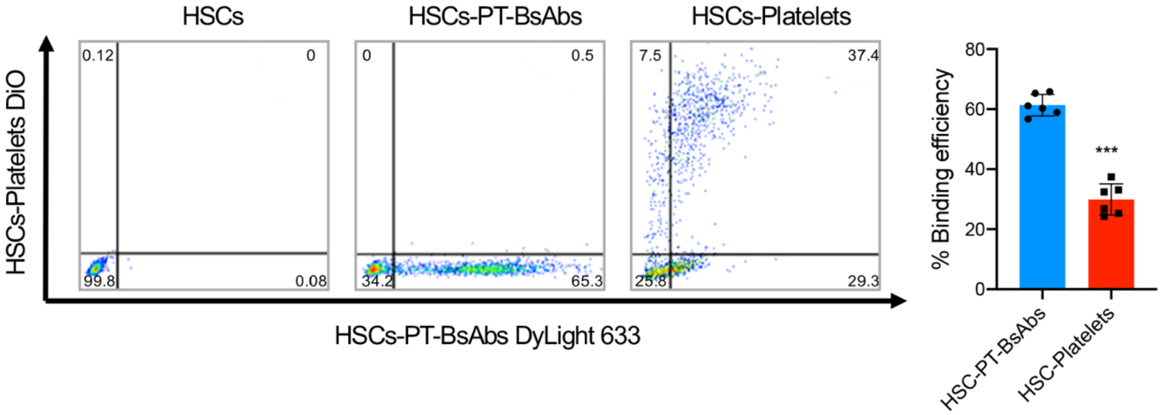

Figure S4. Representative flow cytometry dot plots and quantitative results. Plots indicating unlabeled HSCs (left), PT-BsAbs conjugated HSCs (middle), and HSC-Platelet (HSC-PLT) (right) (n=3). PT-BsAbs were pre-labeled with DyLight 633 while platelets were labeled with DiO.

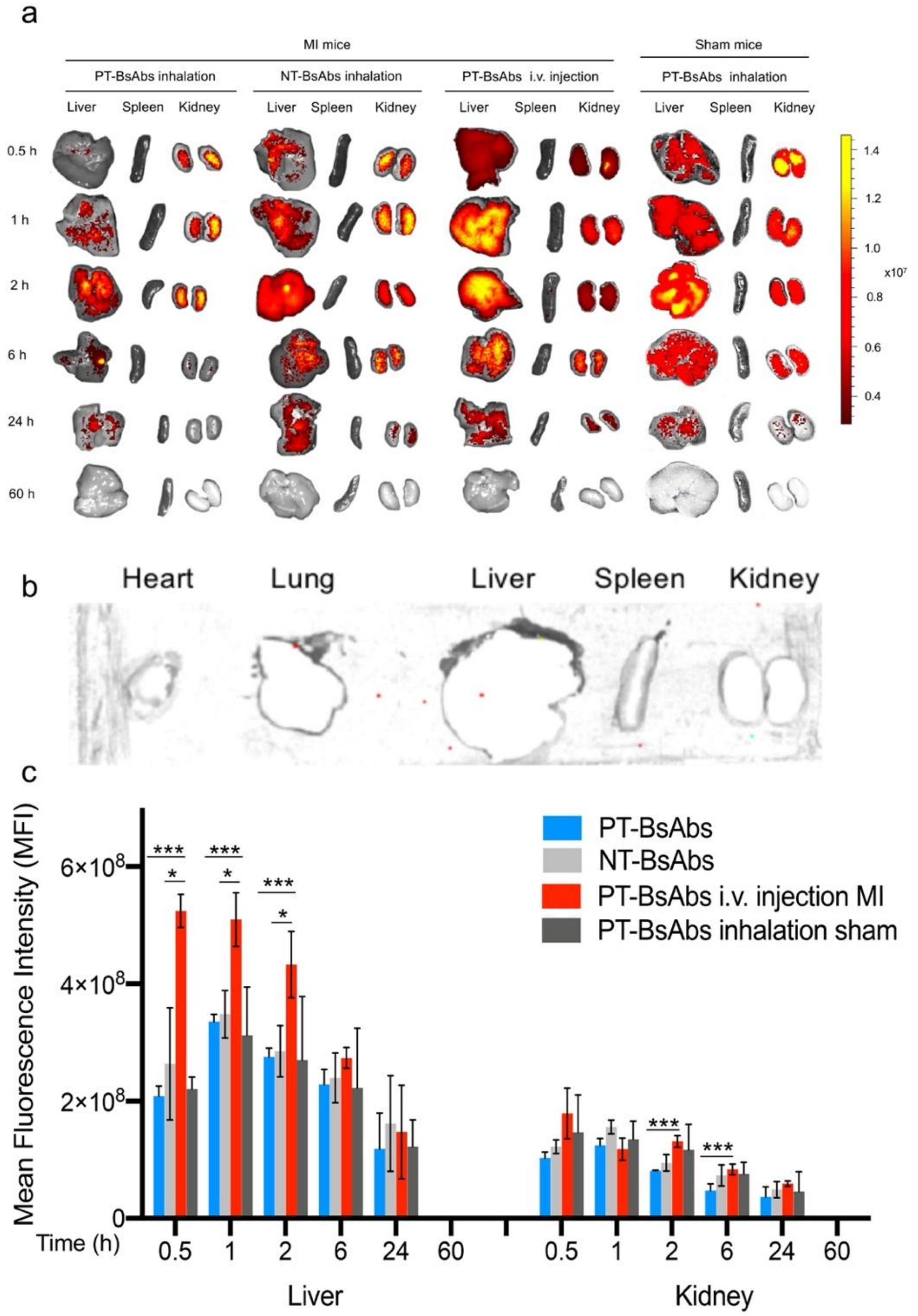

Figure S5. Biodistribution of DyLight 633-labeled BsAbs in liver, spleen and kidney. (a) *Ex vivo* imaging to evaluate the distribution of PT-BsAbs or NT-BsAbs after inhalation, PT-BsAbs after i.v. injection in MI mice, and PT-BsAbs after inhalation in sham mice at different time points. (b) *Ex vivo* imaging of main tissues without fluorescence labeling. (d) Time-course quantification of fluorescence signals from liver and kidney of mice treated with PT-BsAbs via different administrations and inhalant NT-BsAbs. N=3.

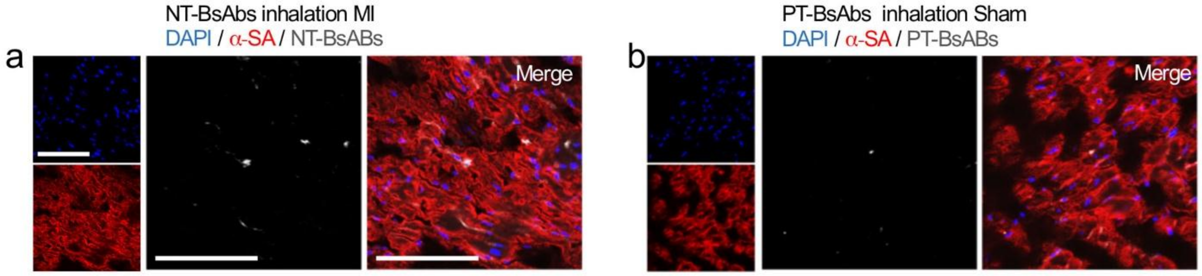

Figure S6. Accumulation of NT-BsAbs or PT-BsAbs in the heart 6 h after treatment. (a) Representative confocal images revealing the NT-BsAb retention in the MI heart 6 h after inhalation administration. (b) Representative confocal images revealing the PT-BsAb retention in the heart of sham mice 6 h after inhalation administration. Cardiomyocytes were stained with alpha sarcomeric actin (α-SA) (red). Nuclei were stained with DAPI (blue). Scale bar, 200 μm. NT-BsAbs or PT-BsAbs were pre-labeled with with DyLight 633 (gray).

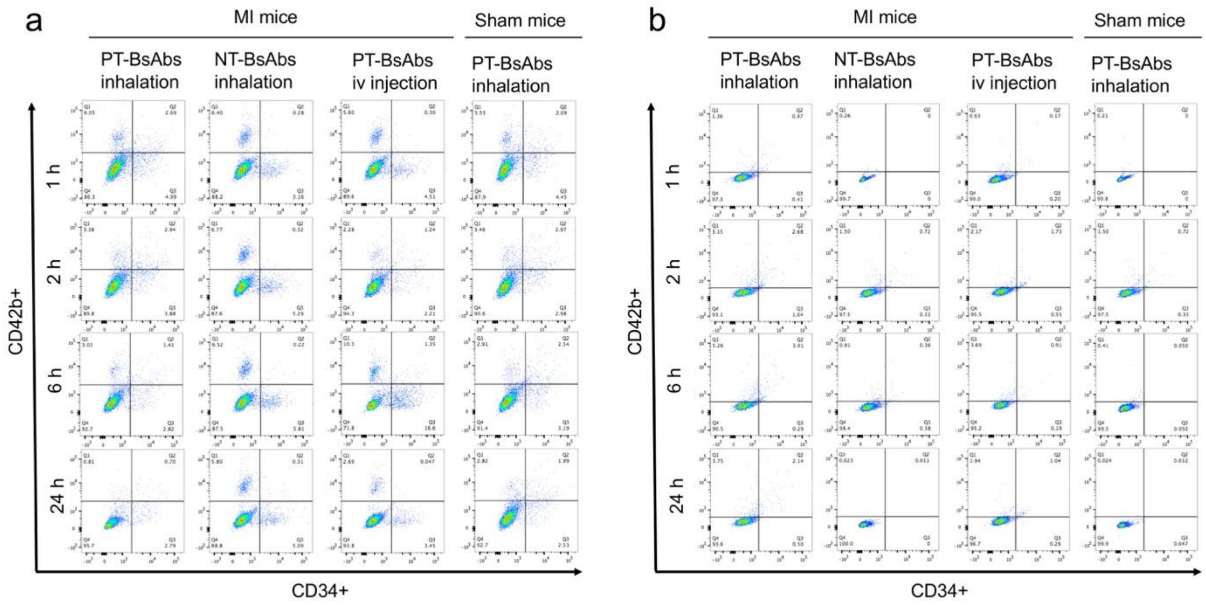

Figure S7. Flow cytometry dot plots. Flow cytometry results of platelet-conjugated HSCs ($CD34^{+}CD42b^{+}$) in the lungs (a) and the heart (b). HSCs and platelets were individually stained with anti-CD34 antibodies and anti-CD42b antibodies, while PT-BsAbs and NT-BsAbs were pre-labeled with DyLight 633.

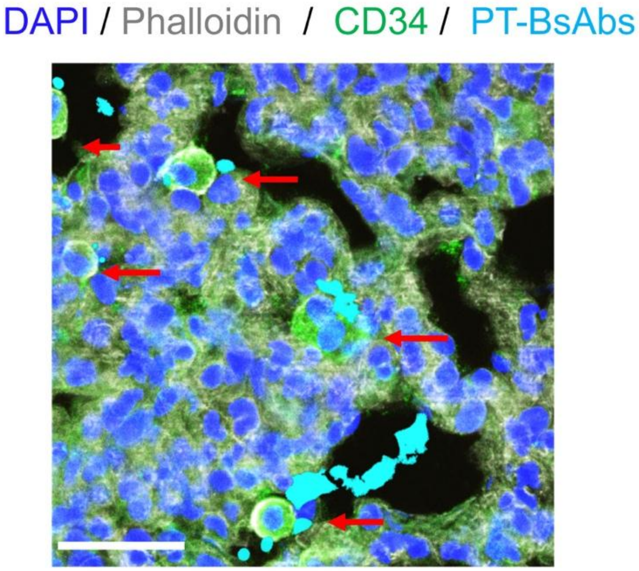

Figure S8. Distribution of HSCs and PT-BsAbs in the lung. Representative confocal images showing conjugation of inhaled PT-BsAbs with HSCs in the lungs of MI mice. Lung cells were stained with anti-Phalloidin antibodies (gray), HSCs were stained with anti-CD34 antibodies (green), nuclei were stained with DAPI (blue). PT-BsAbs were pre-labeled with DyLight 633 (cyan). Scale bar, 25 μm. Red arrowhead indicated HSCs.

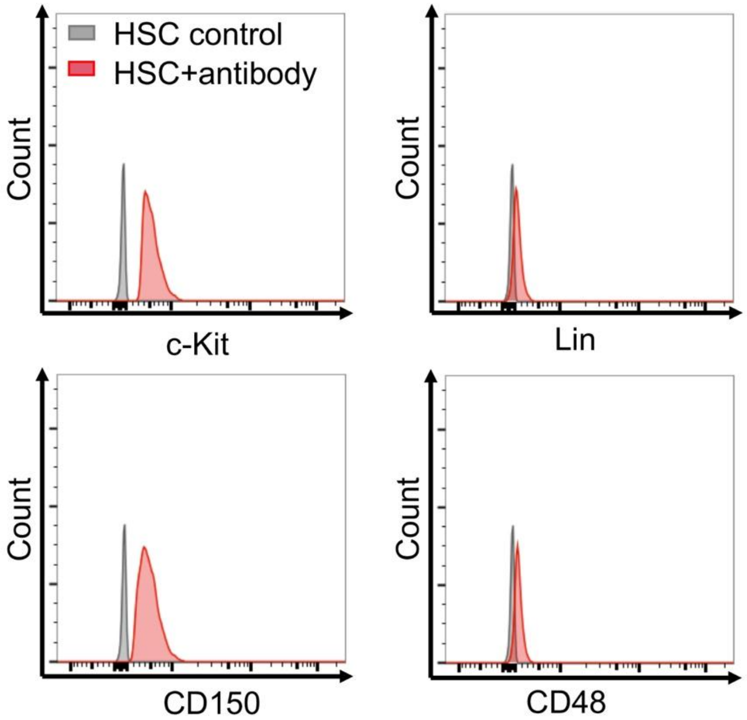

Figure S9. Flow cytometry characterization of CD34$^+$HSCs in the heart.

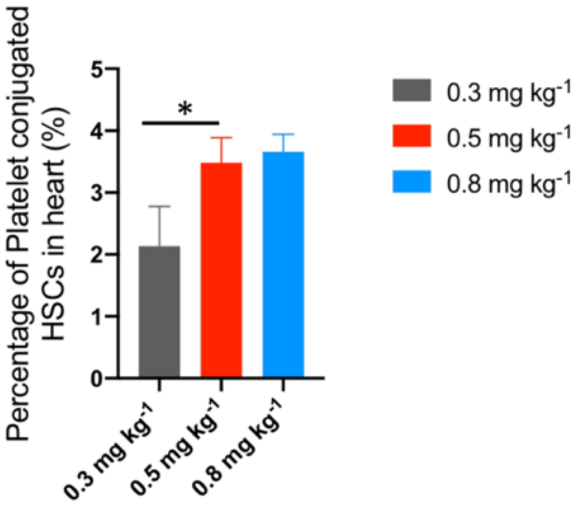

Fig. S10. Flow cytometry quantification results of platelet-conjugated HSCs ($CD34^+CD42b^+$) in the heart 6 h post inhalation of various doses of PT-BsAbs.

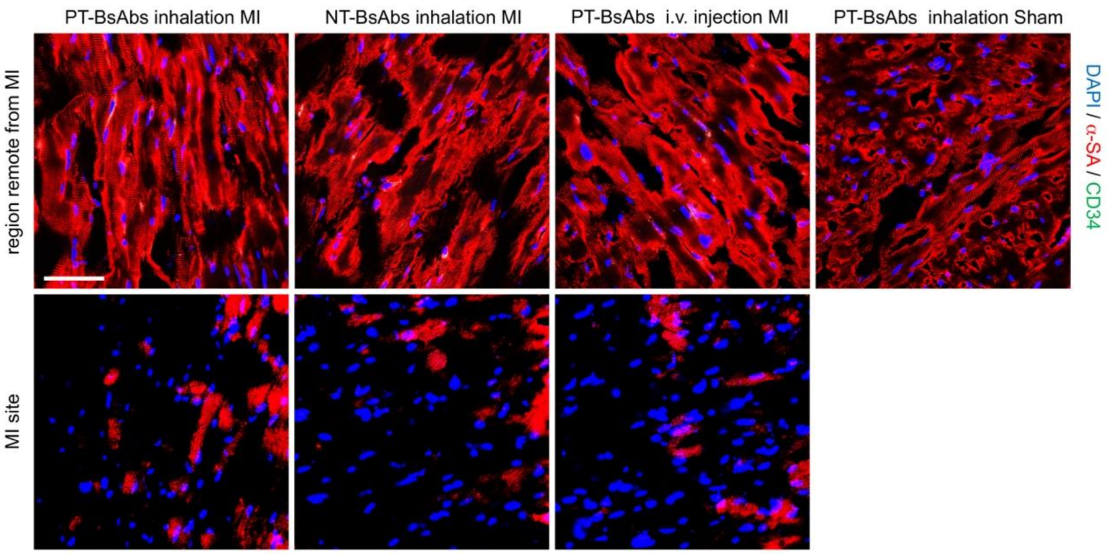

Figure S11. Accumulation of HSCs in healthy and MI areas post-MI. Cardiomyocytes were stained with alpha sarcomeric actin (α-SA) (red). HSCs were stained with CD34 antibody (green). Nuclei were stained with DAPI (blue). Scale bar, 50 µm.

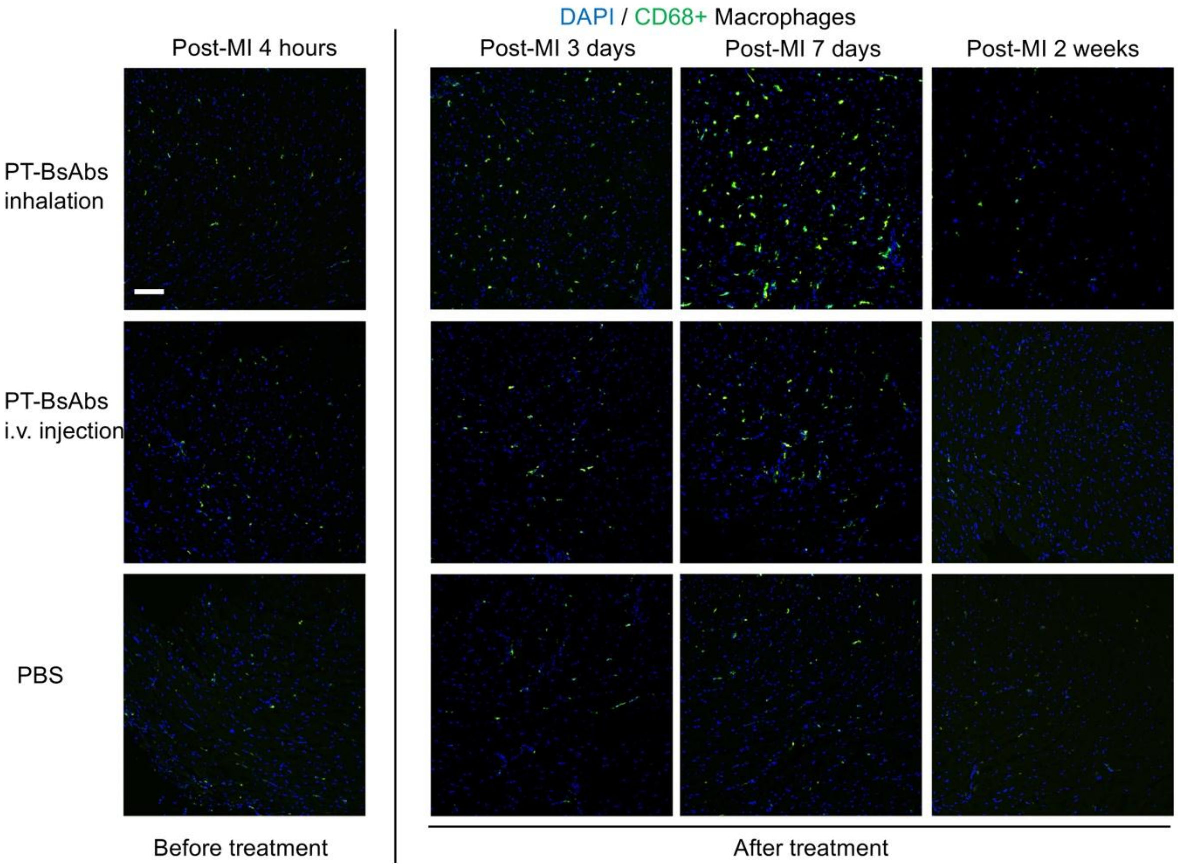

Figure S12. PT-BsAb inhalation increases overall macrophage numbers in the heart. Accumulation of $CD68^+$ macrophages 4 h (before treatments), 3 days, 7 days and 14 days post MI (with treatments). Scale bar, 100 μm.

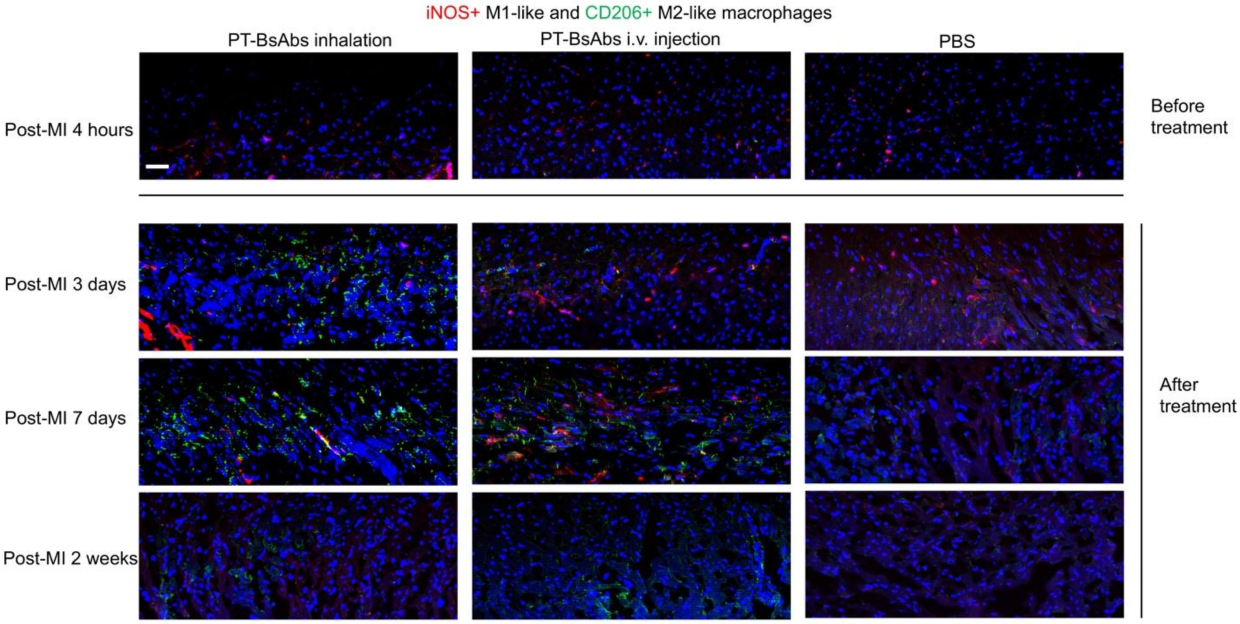

Figure S13. PT-BsAb inhalation increases CD206$^+$ M2-like macrophage percentage and reduces iNOS$^+$ M1-like macrophage percentage in the heart. Accumulation of CD206$^+$ macrophages and iNOS$^+$ macrophages at 4 h (before treatments), 3 days, 7 days and 14 days post MI (with treatments). Scale bar, 50 μm.

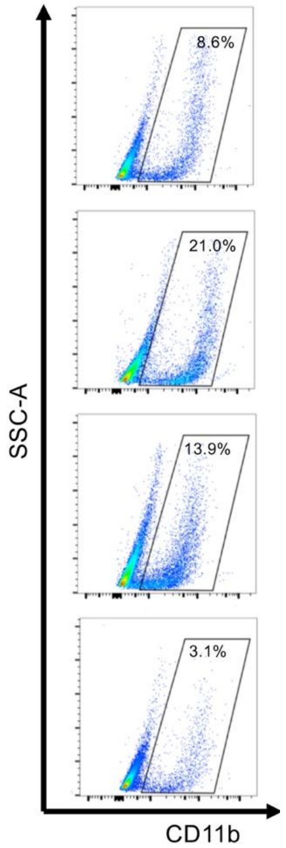

Figure S14. Flow cytometry dot plots of the percentage of CD11b+ cells 4 h post MI (before treatments) or at 3 days, 7 days and 14 days after PT-BsAb inhalation administration.

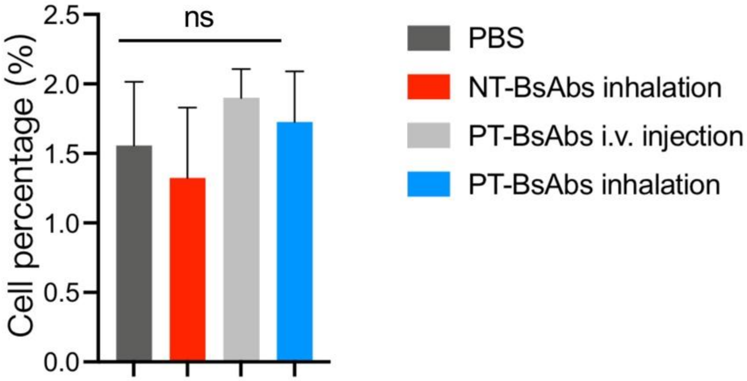

Figure S15. Flow cytometry quantitation of neutrophil accumulation in the heart 7 days post MI in various groups. N=3.

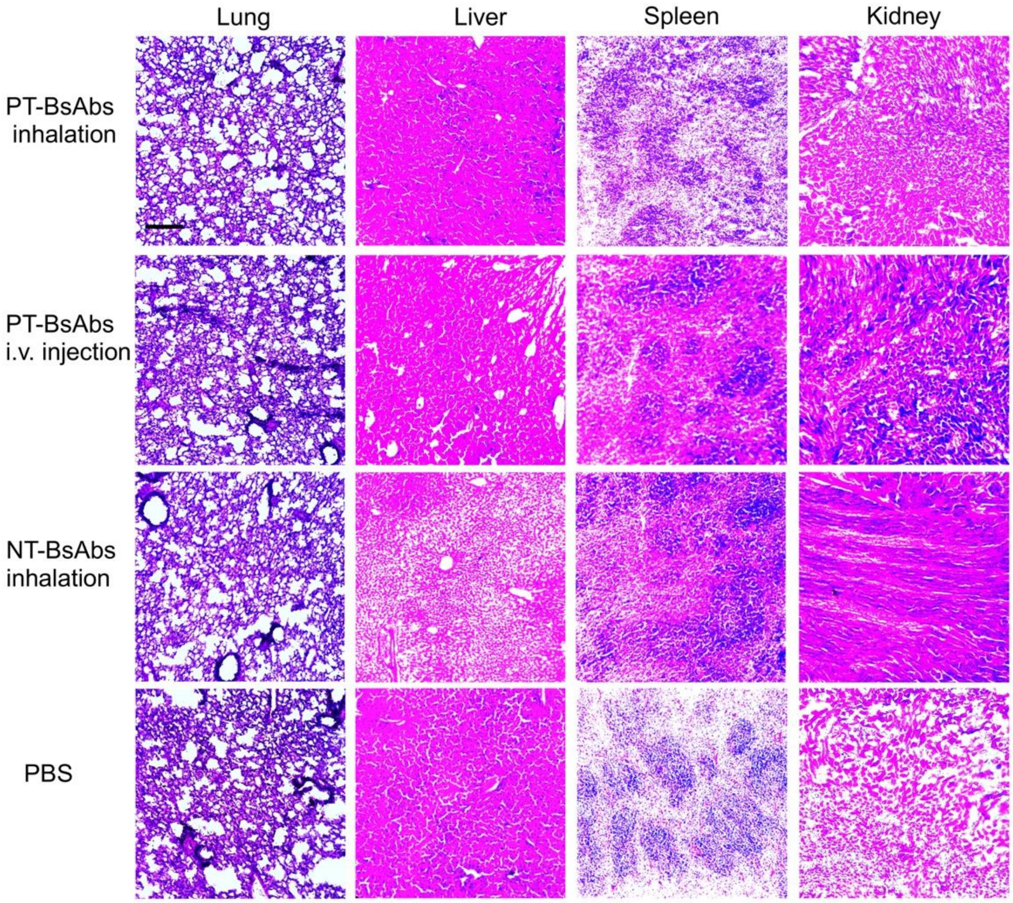

Figure S16. H&E staining of main tissues except heart in mice 21 days after various treatments in MI mice. Scale bar, 200 μm.

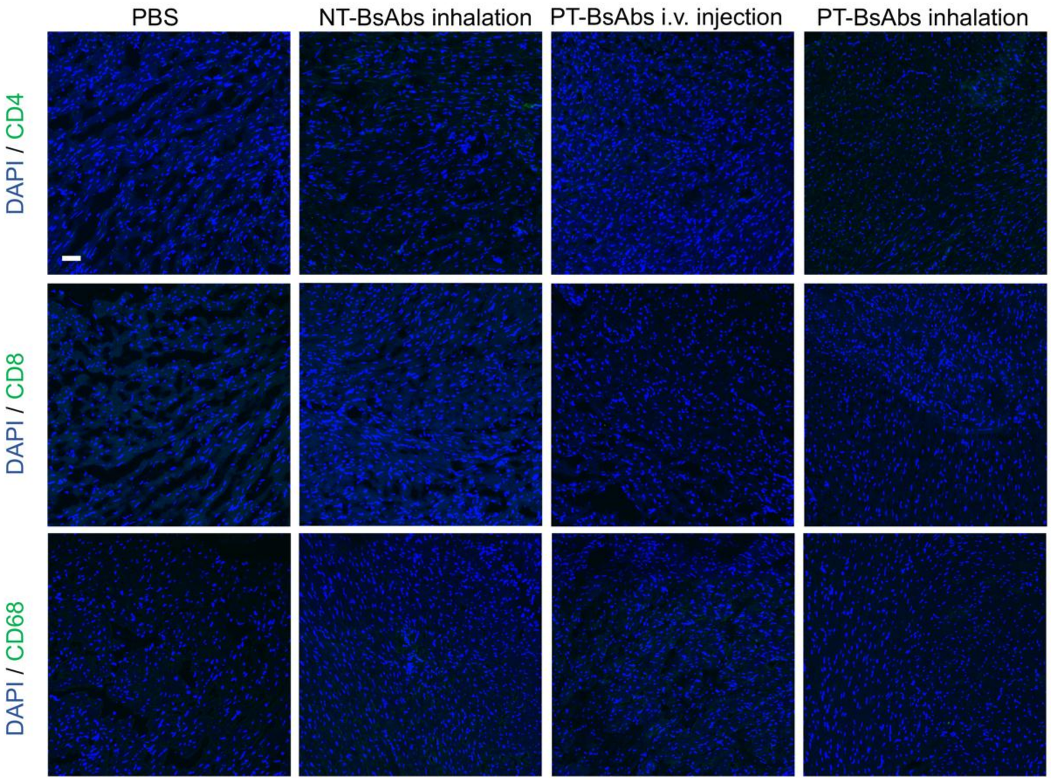

Figure S17. Presence of $CD4^+$ and $CD8^+$ T cells, and $CD68^+$ macrophages in the heart 21 days after various treatments. Scale bar, 60 μm.

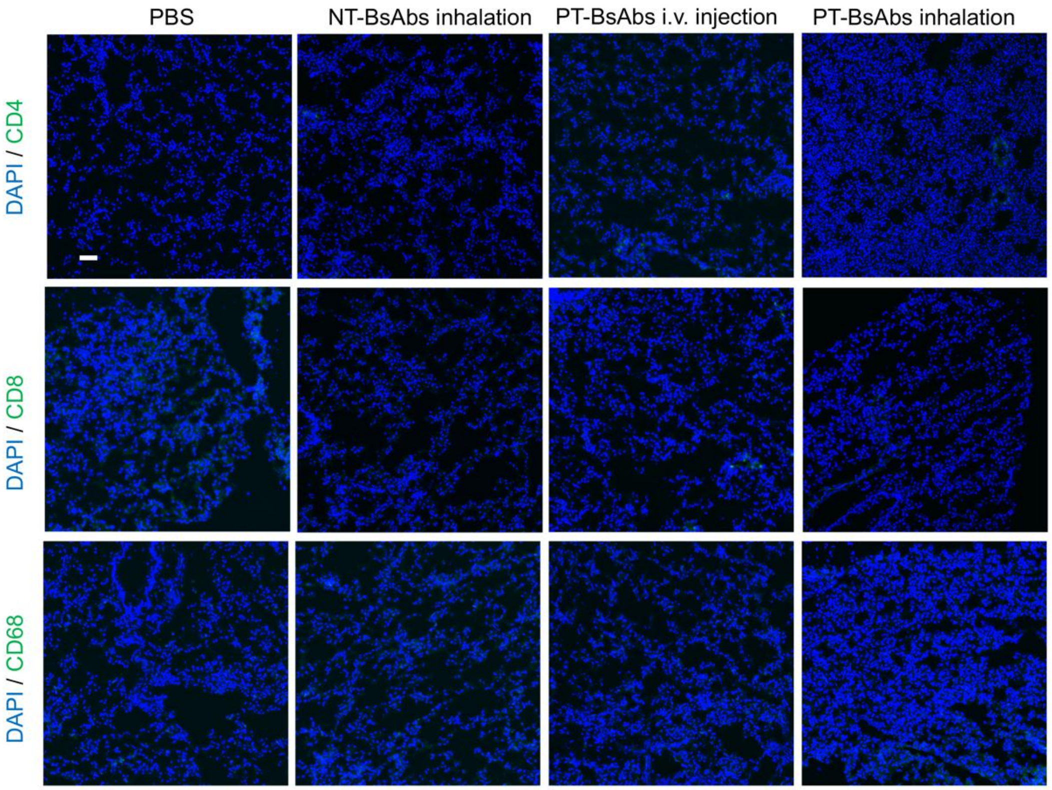

Figure S18. Presence of $CD4^+$ and $CD8^+$ T cells, and $CD68^+$ macrophages in the lung 21 days after various treatments. Scale bar, 60 μm.

Table 1. Antibodies used in the study.

Antibodies	Company	Catalog #	Application
Anti-Rat CD34	BD	BDB553731	BsAbs synthesis/IF
Anti-Mouse CD42b	Santa Cruz Biotechnology	Sc-59052	BsAbs synthesis/IF
Anti-Mouse IgG	Santa Cruz Biotechnology	Sc-2025	BsAbs synthesis
FITC Goat Anti-Rat CD34 antibody	eBioscience	11-0341-82	Cell experiment
FITC Goat Anti-Mouse CD42b antibody	eBioscience	11-0429-42	Cell experiment
Alexa Fluor® 594 Anti-Phalloidin	Abcam	ab176757	IF
Goat Anti-Rabbit vWF	Abcam	ab111713	IF
Goat Anti-Rabbit α-SA	Abcam	Ab72592	IF
Goat Anti-Rabbit ki67	Abcam	Ab15580	IF
Goat Anti-Rabbit Caspase-3	Abcam	Ab13847	IF
Goat Anti-Rabbit Cleaved PARP	Abcam	Ab32064	IF
Goat Anti-Rabbit α-SMA	Abcam	Ab5694	IF
Goat Anti-Rabbit CD68	Abcam	ab125212	IF
Goat Anti-Rabbit iNOS	Abcam	Ab15323	IF
Goat Anti-Mouse CD206	Abcam	Ab64693	IF
Goat Anti-Rabbit CD8	Abcam	Ab217344	IF
Goat Anti-Rabbit CD4	Abcam	Ab237722	IF
FITC Anti-Mouse F4/80	eBioscience	11-4801-82	Flow Cytometry
PE Anti-Mouse CD11b	eBioscience	12-0112-82	Flow Cytometry
PE/Cy7 Anti-Mouse CCR2	BioLegend	150611	Flow Cytometry
Alexa Fluor® 488 Goat Anti-Rabbit IgG H&L	Abcam	Ab150077	IF
Alexa Fluor® 647 Goat Anti-Mouse IgG H&L	Abcam	Ab150115	IF
Alexa Fluor® 647 Goat Anti-Rabbit IgG H&L	Abcam	Ab150083	IF
Alexa Fluor® 488 Goat Anti-Mouse IgG H&L	Abcam	Ab150113	IF
